# Overexpression of *GmMYB14* improves high‐density yield and drought tolerance of soybean through regulating plant architecture mediated by the brassinosteroid pathway

**DOI:** 10.1111/pbi.13496

**Published:** 2020-11-23

**Authors:** Limiao Chen, Hongli Yang, Yisheng Fang, Wei Guo, Haifeng Chen, Xiaojuan Zhang, Wenjun Dai, Shuilian Chen, Qingnan Hao, Songli Yuan, Chanjuan Zhang, Yi Huang, Zhihui Shan, Zhonglu Yang, Dezhen Qiu, Xiaorong Liu, Lam‐Son Phan Tran, Xinan Zhou, Dong Cao

**Affiliations:** ^1^ Key Laboratory of Biology and Genetic Improvement of Oil Crops Ministry of Agriculture and Rural Affairs Oil Crops Research Institute Chinese Academy of Agricultural Sciences Wuhan China; ^2^ The Industrial Crop Institute Shanxi Academy of Agricultural Sciences Taiyuan China; ^3^ Institute of Genomics for Crop Abiotic Stress Tolerance Department of Plant and Soil Science Texas Tech University Lubbock TX USA; ^4^ Stress Adaptation Research Unit RIKEN Center for Sustainable Resource Science Tsurumi Yokohama Japan

**Keywords:** plant architecture, drought tolerance, MYB transcription factor, soybean, yield, brassinosteroids

## Abstract

MYB transcription factors (TFs) have been reported to regulate the biosynthesis of secondary metabolites, as well as to mediate plant adaption to abiotic stresses, including drought. However, the roles of MYB TFs in regulating plant architecture and yield potential remain poorly understood. Here, we studied the roles of the dehydration‐inducible *GmMYB14* gene in regulating plant architecture, high‐density yield and drought tolerance through the brassinosteroid (BR) pathway in soybean. GmMYB14 was shown to localize to nucleus and has a transactivation activity. Stable *GmMYB14*‐overexpressing (*GmMYB14‐OX*) transgenic soybean plants displayed a semi‐dwarfism and compact plant architecture associated with decreased cell size, resulting in a decrease in plant height, internode length, leaf area, leaf petiole length and leaf petiole angle, and improved yield in high density under field conditions. Results of the transcriptome sequencing suggested the involvement of BRs in regulating *GmMYB14‐OX* plant architecture. Indeed, *GmMYB14‐OX* plants showed reduced endogenous BR contents, while exogenous application of brassinolide could partly rescue the phenotype of *GmMYB14‐OX* plants. Furthermore, GmMYB14 was shown to directly bind to the promoter of *GmBEN1* and up‐regulate its expression, leading to reduced BR content in *GmMYB14‐OX* plants. *GmMYB14‐OX* plants also displayed improved drought tolerance under field conditions. *GmBEN1* expression was also up‐regulated in the leaves of *GmMYB14‐OX* plants under polyethylene glycol treatment, indicating that the *GmBEN1*‐mediated reduction in BR level under stress also contributed to drought/osmotic stress tolerance of the transgenic plants. Our findings provided a strategy for stably increasing high‐density yield and drought tolerance in soybean using a single TF‐encoding gene.

## Introduction

Soybean (*Glycine max*) is an economically relevant legume crop, which has been grown worldwide for edible oil, protein sources and biodiesel (www.soystats.com). Plant architecture is an essential trait for the development of high‐yield cultivars in soybean, which can be achieved by modulating genes that control stem growth habit, node number, plant height (PH), internode length (IL), branch number and leaf size and shape (Bao *et al*., 2019; Sun *et al*., 2019). Over the last few decades, great efforts have been made to improve soybean yields through the stem growth habit‐based selection for semi‐dwarf phenotype, which is one of the most important traits for the improvement of lodging tolerance and yield of this important crop (Liu *et al*., 2010; Ping *et al*., 2014; Tian *et al*., 2010). In cereal crops, plant varieties that have architecture with smaller leaf angle can be cultivated under higher plant density, thereby showing increased grain yields (Hake and Richardson, 2019; Tian *et al*., 2019). For instance, the changes in leaf angles that allowed planting maize (*Zea mays*) in high density have boosted the maize yields (Tian *et al*., 2019). Genetic and functional studies have suggested that genes involved in brassinosteroid (BR) biosynthesis play important roles in regulating leaf angles in rice (*Oryza sativa*) (Je *et al*., 2010; Sun *et al*., 2015), maize (Ren *et al*., 2019; Strable *et al*., 2017; Tian *et al*., 2019) and wheat (*Triticum aestivum*) (Liu *et al*., 2019). Additionally, Gao *et al*. (2017) revealed that *G. max INCREASED LEAF PETIOLE ANGLE 1* (*GmILPA1*), encoding a subunit of the anaphase‐promoting complex, controlled the leaf petiole angle in soybean by regulating the establishment of pulvinus through its function in promoting cell proliferation (Gao *et al*., 2017). However, gene regulatory network involved in regulating compact plant architecture and yield potential remains poorly understood at a comprehensive level, particularly in soybean.

In plants, several transcription factors (TFs), including SQUAMOSA PROMOTER‐BINDING PROTEIN‐LIKE (SPL) (Bao *et al*., 2019; Jiao *et al*., 2010; Liu *et al*., 2019; Miura *et al*., 2010; Sun *et al*., 2019), RELATED TO ABI3/VP1‐LIKE 1 (RAVL1) (Je *et al*., 2010; Tian *et al*., 2019), WRINKLED1 (Guo *et al*., 2020) and BRANCHED 1 (BRC1) TFs (van Rongen *et al*., 2019; Seale *et al*., 2017; Shen *et al*., 2019), have a great role in regulating plant architecture. In addition, the *BLIND* genes, encoding proteins with an R2R3‐MYB domain, have been reported to regulate the lateral meristems in tomato (*Solanum lycopersicum*) (Schmitz *et al*., 2002) and *Arabidopsis thaliana* (Muller *et al*., 2006). Recently, the expression of *GmMYB181*, which also encodes an R2R3‐MYB TF, was found to be enriched in flowers, and ectopic expression of *GmMYB181* in *Arabidopsis* increased lateral branches and reduced plant height, as well as altered the fruit size and floral organs of the transgenic plants (Yang *et al*., 2018). However, the roles of *MYB* genes in regulating plant architecture and yield potential remain poorly understood.

The MYB proteins form one of the largest TF families in plants (Du *et al*., 2015; Mochida *et al*., 2010). A considerable number of *MYB* genes have been reported to regulate the biosynthesis of secondary metabolites, including proanthocyanidins and anthocyanins (Jun *et al*., 2015), chlorophylls and carotenoids (Ampomah‐Dwamena *et al*., 2019), lignins (Chezem *et al*., 2017), flavonoids (Ma *et al*., 2018) and isoflavonoids (Bian *et al*., 2018), as well as to mediate plant adaption to abiotic stresses, including drought and cold (An *et al*., 2018; Chezem *et al*., 2017; Ding *et al*., 2009; Wang *et al*., 2007a,b; Xie *et al*., 2018). Previously, we reported that the *Glyma19g164600* (*GmMYB14*) gene, which encodes an R2R3‐MYB TF, was up‐regulated in soybean by dehydration, suggesting its potential involvement in regulation of soybean response to drought (Chen *et al*., 2013a).

In the current study, we further studied the functions of *GmMYB14* gene in more detail by characterizing its expression patterns, subcellular localization and transactivation activity, and by developing stable transgenic soybean lines overexpressing *GmMYB14* (*GmMYB14‐OX*) to dissect the roles of this TF in regulation of plant architecture, high‐density yield and drought tolerance in soybean. We showed that stable overexpression of *GmMYB14* resulted in altered plant architecture with decreases in PH, IL, leaf area, leaf petiole length and leaf petiole angle, but increases in yield component traits such as pod number per plant (PN), seed number per plant (SN) and seed weight per plant (SW) under high density in field conditions. These ideal changes in plant architecture of *GmMYB14‐OX* lines were attributed to the decreased activity of the BR pathway. Importantly, *GmMYB14‐OX* lines displayed enhanced drought tolerance in the field test. The molecular mechanisms underlying *GmMYB14*‐mediated regulation of plant architecture and drought tolerance were also elucidated by using RNA sequencing (RNA‐seq). The semi‐dwarf phenotype of *GmMYB14‐OX* plants might be explained by the decrease in cell size as indicated by an anatomical structure analysis. Our findings will facilitate the efforts to alter plant architecture for improvement of yield and drought tolerance in soybean.

## Results

### Characterization of the *GmMYB14* gene in soybean

**Figure 1:**
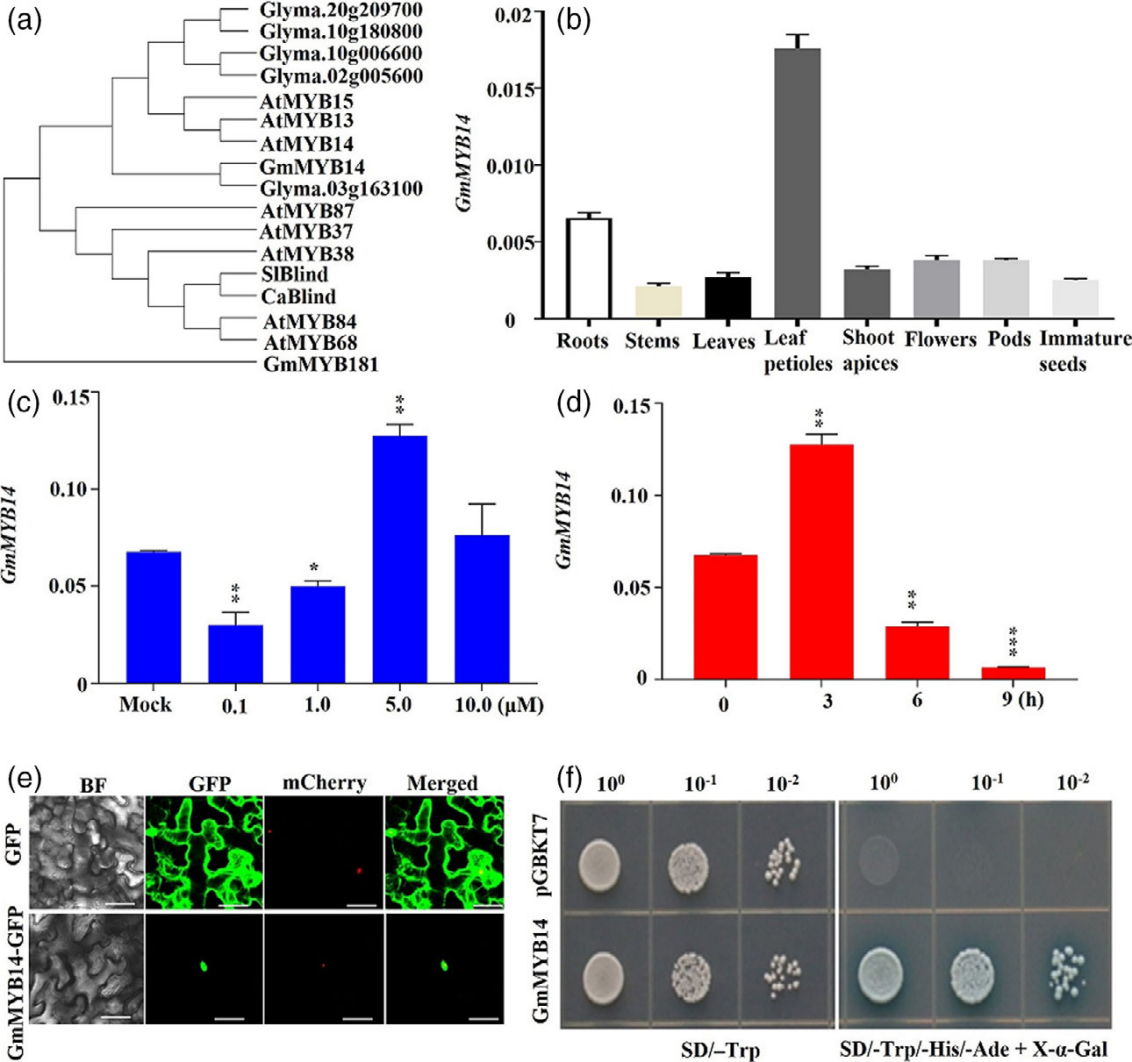
Phylogenetic relationship, gene expression analysis, localization and transactivation assay of the GmMYB14 transcription factor. (a) Phylogenetic analysis of GmMYB14 and its orthologs in soybean (Glyma.03G163100, Glyma.20G209700, Glyma.10G180800, Glyma.10G006600 and Glyma.02G005600), *Arabidopsis thaliana* (AtMYB13, AtMYB14 and AtMYB15) and other MYB proteins in soybean (GmMYB181), *Capsicum annuum* (CaBlind) and *Solanum lycopersicum* (SlBlind) involved in regulating plant architecture. (b) Expression of *GmMYB14* in different organs. Relative expression levels were normalized to the expression level of *GmActin*. (c) Expression of *GmMYB14* in leaves after treatment with different concentrations of brassinolide (BL) for 3 h (*n* = 3 biological repeats). (d) Expression of *GmMYB14* in leaf samples after treatment with 5 μM BL for different time periods (*n* = 3 biological repeats). (e) GmMYB14 is localized to the nucleus in tobacco epidermal cells. The fluorescences of GmMYB14‐GFP and GFP (negative control) were visualized with a high‐resolution laser confocal microscope at 488 nm. BF, bright field; mCherry, FIBRILLARIN2‐mCherry nucleolar marker; bar = 20 µm. (f) Transactivation assay of GmMYB14 in yeast cells harbouring ‘pGBKT7:GmMYB14’ construct. Transformed yeast cells harbouring ‘pGBKT7’ vector were used as a negative control. In each panel, the yeast cells harbouring the indicated plasmid combinations were grown on either the nonselective (e.g. SD/‐Trp) or selective (e.g. SD/‐Trp/‐His/‐Ade medium containing X‐α‐Gal) medium that allowed the α‐galactosidase activity assay (X‐α‐Gal staining). Decreasing cell densities in the dilution series are illustrated by 10^0^, 10^−1^and 10^−2^. Statistically significant difference between each BL treatment and mock is marked with asterisk(s) (*P < 0.05; **P < 0.01; and ***P < 0.001; Student's *t*‐test)

Phylogenetic analysis showed that the *Glyma19g164600* gene encodes a MYB protein, assigned GmMYB14, because it displayed the highest homology to the *Arabidopsis* AtMYB14 with a typical R2R3‐MYB domain (Chen *et al*., 2013b) (Figure 1a). Expression analysis of the *GmMYB14* gene in different organs revealed that it was expressed in all eight examined organs, displaying the highest transcript levels in leaf petioles followed by roots, pods, flowers, shoot apices, leaves, immature seeds and stems (Figure 1b). The expression of *GmMYB14* was also induced by brassinolide (BL) treatment in leaves (Figure 1c and d). Particularly, *GmMYB14* expression was highly induced in leaves after treatment with 5 μm BL (Figure 1c). Furthermore, under the condition of 5 μm BL treatment, the transcript levels of *GmMYB14* in leaves were the highest at 3 h (Figure 1d).

To study the subcellular localization of the GmMYB14 protein, a GmMYB14 fusion protein construct was generated by inserting the coding sequence (CDS) of *GmMYB14* in a way to fuse the GmMYB14 in frame with the N‐terminus of the green fluorescent protein (GFP). As expected, GmMYB14 was localized to the nucleus in tobacco (*Nicotiana tabacum*) epidermal cells (Figure 1e). In addition, results obtained from a transactivation assay indicated that GmMYB14 has transactivation activity, as the GAL4 DNA‐binding domain:GmMYB14 fusion could activate the *HIS3*, *ADE2* and *MEL1* reporter genes in the transformed yeast cells, resulting in their growth and α‐Gal activity on the selective synthetic defined (SD)/‐Trp/‐His/‐Ade medium containing X‐α‐Gal (Figure 1f).

### Overexpression of *GmMYB14* caused semi‐dwarf plant architecture in transgenic soybean

To investigate whether *GmMYB14* has a role in the regulation of plant architecture, the CDS of *GmMYB14* was placed under the control of the cauliflower mosaic virus CaMV *35S* promoter, and the construct was introduced into soybean for generation of stable *GmMYB14‐OX* transgenic soybean lines (Figure S1a). We obtained five independent homozygous *GmMYB14‐OX* lines, named OX1, OX7, OX9, OX10 and OX12, which showed one (OX1 and OX12), two (OX7 and OX9) or three (OX10) copies of the transgene as evidenced by the results of a southern blot analysis (Figure S1b). At the V5 vegetative stage, OX1, OX9 and OX12 plants showed semi‐dwarf phenotype, while OX7 and OX10 displayed similar phenotype as wild‐type (WT) plants (Figure S1c, only the image of OX7 was shown as a representative phenotype). These phenotypic data showed a positive correlation with the overexpression levels of the *GmMYB14* gene (Figure S1d), suggesting that the semi‐dwarfism was resulted from the overexpression of *GmMYB14*. Thus, OX1, OX9 and OX12 lines were used for further studies in this research.

**Figure 2:**
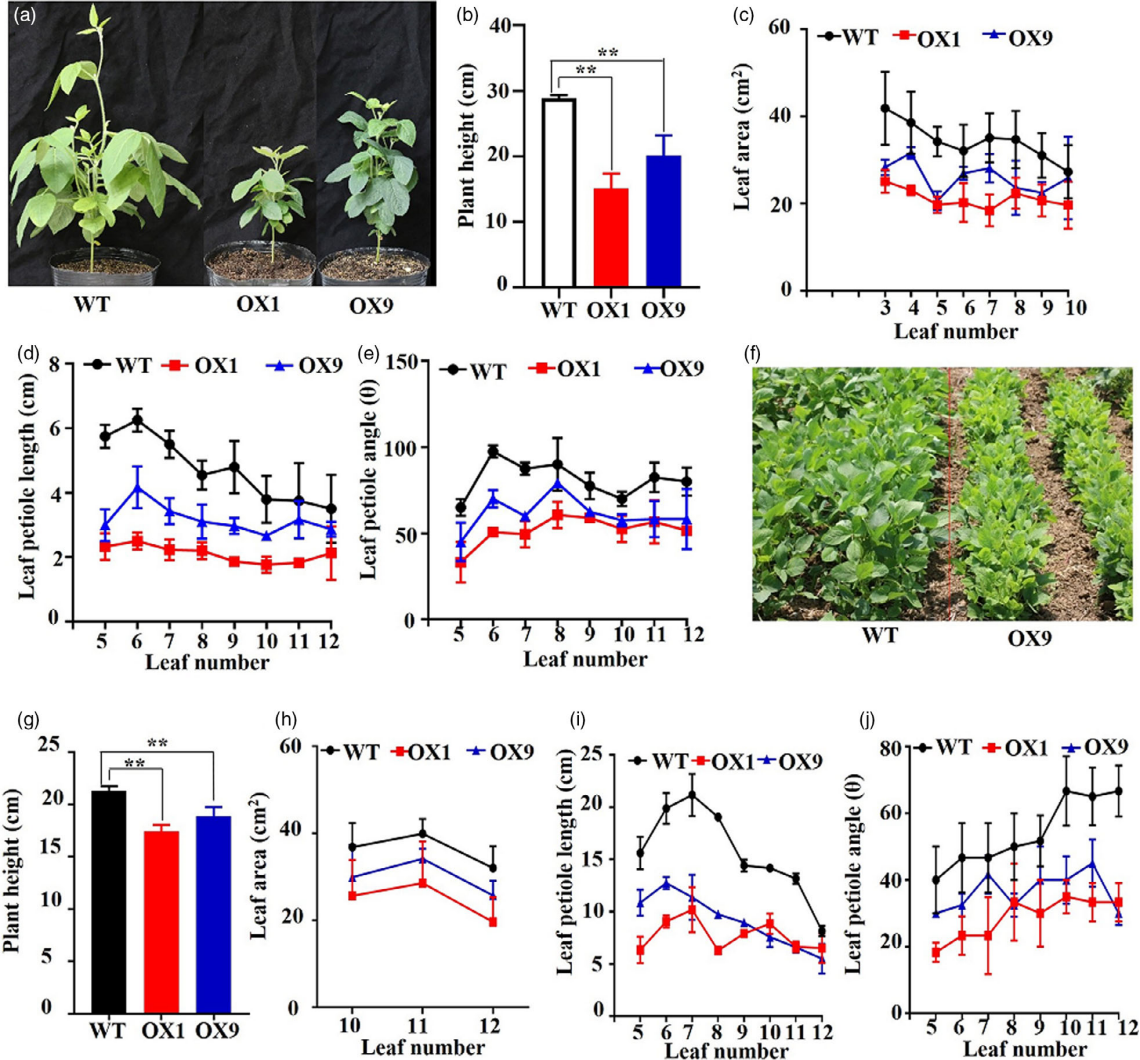
Comparison of the phenotypes of *GmMYB14*‐*OX* transgenic and wild‐type (WT) plants at vegetative and reproductive growth stages. (a) Representative pictures showing plant architecture of 30‐day‐old T3‐generation OX1, OX9 and WT plants at the V6 stage under controlled growth conditions. (b) Plant height, (c) leaf area, (d) leaf petiole length and (e) leaf petiole angle of 30‐day‐old T3‐generation OX1, OX9 and WT plants grown under controlled growth conditions. Data shown are means and standard deviations of six plants (*n* = 6 plants/line). (f) Representative pictures showing plant architecture of 38‐day‐old T3‐generation OX9 and WT plants at the reproductive R1 stage under field conditions in 2018, with a distance of 40 cm between two rows and a distance of 10‐cm interval distance between two plants within a row. (g) Plant height, (h) leaf area, (i) leaf petiole length and (j) leaf petiole angle of 38‐day‐old T3‐generation OX1, OX9 and WT plants (R1 stage) grown under field conditions in 2018, with a distance of 40 cm between rows and a distance of 10‐cm interval distance between two plants within a row. Data shown are means and standard deviations of five plants (*n* = 5 plants/line). Statistically significant differences between each transgenic line and WT are marked with asterisk(s) (**P < 0.01; Student's *t*‐test).

Detailed phenotypic analysis of OX1 and OX9 grown under the controlled growth conditions showed a remarkable change in plant architecture with decreases in PH, leaf area, leaf petiole length and leaf petiole angle at the V6 stage, when compared with WT plants (Figure 2a–e). OX1 and OX9 also displayed shorter primary root lengths than WT plants grown under the hydroponic conditions (Figure S2). We, next, tested whether OX1 and OX9 lines could regulate plant architecture under field conditions. Similar to the results obtained under the controlled growth conditions (Figure 2a–e), the field‐grown transgenic OX1 and OX9 plants also showed an altered plant architecture at the R1 stage with decreased PH, leaf area, leaf petiole length and leaf petiole angle in comparison with WT plants (Figure 2f–j).

**Figure 3:**
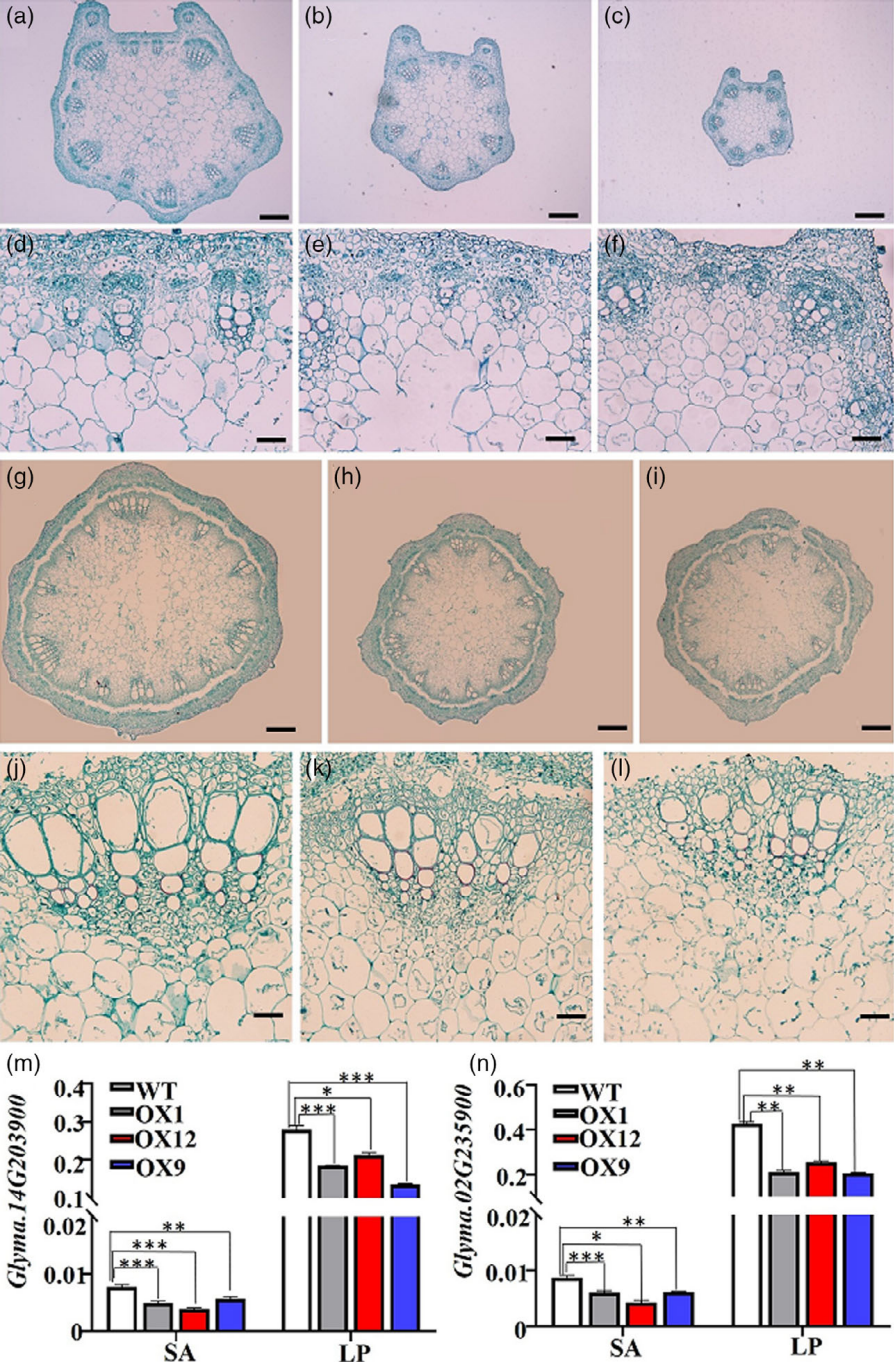
Overexpression of *GmMYB14* decreased cell size in transgenic soybean plants at vegetative growth stage. (a–c) Transection of leaf petioles of 30‐day‐old wild‐type (WT) plants (a), T2‐generation OX9 (b) and OX1 (c). Bar = 200 µm. (d–f) Partial magnification of (a–c) with bar = 50 µm. (g–i) Transection of stems of 30‐day‐old WT plants (g), T2‐generation OX9 (h) and OX1 (i). Bar = 200 µm. (j–l) Partial magnification of (g–i) with bar = 50 µm. The leaf petioles and stems between the fifth and sixth nodes were collected from the V6‐stage OX1, OX9 and WT plants. (m–n) The expression level of *Glyma.14G203900* (m) and *Glyma.02G235900* (n) in OX1, OX9, OX12 and WT plants. Shoot apices (SA) and leaf petioles (LP) were harvested from the seedlings at 12^th^ day after sowing for qRT‐PCR. Data shown are means and standard deviations (*n* = 3 biological replicates). Statistically significant differences between transgenic and WT plants in terms of gene expression are marked with asterisks (*P < 0.05, **P < 0.01, ***P < 0.001; Student's*t*‐test).

An analysis of the anatomical structure of stems and leaf petioles revealed that the cell sizes decreased in the OX1 and OX9 transgenic plants, particularly in the case of leaf petioles, as compared with that of WT plants (Figure 3a–l). Previously, Yin *et al*. (2019) suggested that cell elongation rate is positively correlated with the expression levels of genes encoding cell wall‐modifying enzymes, such as extensins, expansins and xyloglucan endotransglucosylases. We selected two genes (*Glyma.14G203900* and *Glyma.02G235900*) encoding expansins as an example to test such correlation in the transgenic and WT plants using quantitative real‐time PCR (qRT‐PCR). Figure 3m–n showed that the expression of these two genes was repressed in shoot apices and leaf petioles of the three transgenic lines (OX1, OX9 and OX12), compared with WT plants. These results collectively demonstrated that *GmMYB14* plays a critical role in regulating the plant architecture in soybean by affecting the cell growth rate, perhaps through modulation of the expression of genes encoding cell wall‐modifying enzymes.

### *GmMYB14‐OX* plants showed enhanced yields under field conditions

Next, to investigate whether the overexpression of *GmMYB14* altered yields of *GmMYB14‐OX* lines grown under field conditions, we measured various agronomic and yield‐related traits of the transgenic plants grown in Hanchuan Transgenic Biosafety Station (HTBS) at the 10‐cm interval distance in 2018 and 2019. At the harvest stage, the homozygous T3‐generation OX9 plants grown in 2018 displayed decreases in PH and IL, but increases in node number on main stem (NN), branch number on main stem (BN), PN, SN and SW (e.g. yield per plant), while no difference in 100‐seed weight was observed, in relation to WT plants (Figure S3a–h). Similar tendency was found with the results recorded for PH, IL, NN, BN, PN, SN, SW and 100‐seed weight of homozygous T3‐generation OX1 and T4‐generation OX9 plants that were cultivated in 2019 under field conditions (Figure S3i–q).

**Figure 4:**
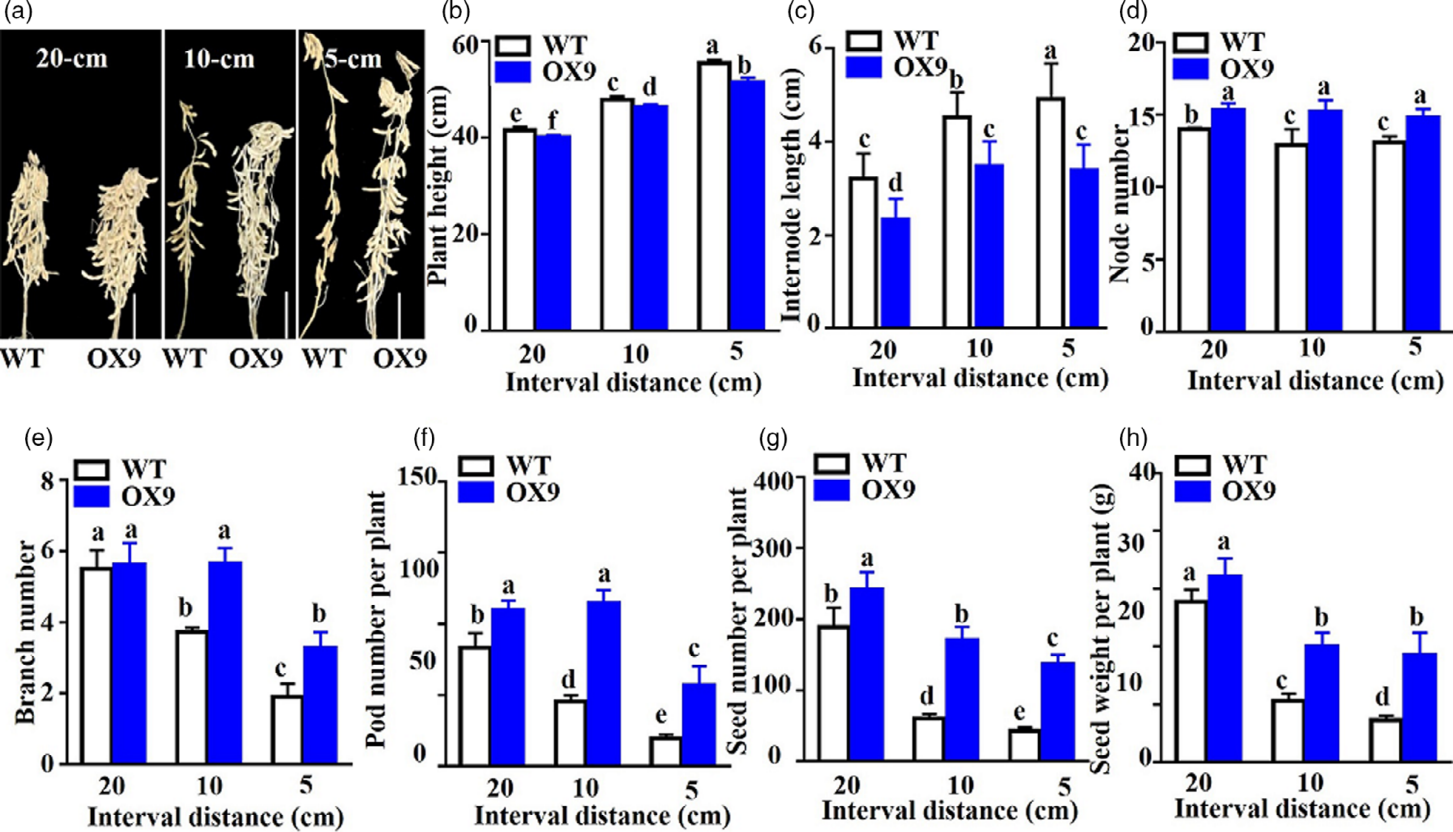
*GmMYB14*‐*OX* transgenic soybean plants produced higher yield under high planting densities. (a) Representative pictures showing plant architecture (bar = 10 cm), (b) plant height, (c) internode length, (d) node number, (e) branch number, (f) pod number per plant, (g) seed number per plant and (h) seed weight per plant of T4‐generation OX9 and WT plants grown under field conditions at three plant densities (5‐, 10‐ and 20‐cm interval distances between two plants within a row, and 40‐cm distance between two rows) in 2019. Data shown are means and standard deviations (*n* = 3 replicates; 5 plants/genotype/replicate). Significant differences were shown by different letters following Duncan's multiple‐range test (*P* < 0.05).

To investigate whether the OX plants with semi‐dwarf plant architecture could enhance soybean yields when they were grown under high density, we performed a field experiment and measured the agronomic and yield traits of the homozygous T4‐generation OX9 plants in HTBS under three plant densities (5‐, 10‐ and 20‐cm interval distances between two plants within a row) in 2019. In comparison with WT, similar differential results were recorded in PH, IL, NN, BN, PN, SN and SW of OX9 plants at all three plant densities (Figure 4). In particular, the SW of the OX9 line increased by 15.9, 93.0 and 156.0% at the density of 20‐, 10‐ and 5‐cm interval distances, respectively, over that of WT (Figure 4h). These data indicated that the *GmMYB14* had a role in regulating the plant architecture and improving yield potential under the growth conditions of high density.

### Overexpression of *GmMYB14* enhanced drought tolerance in transgenic soybean

**Figure 5:**
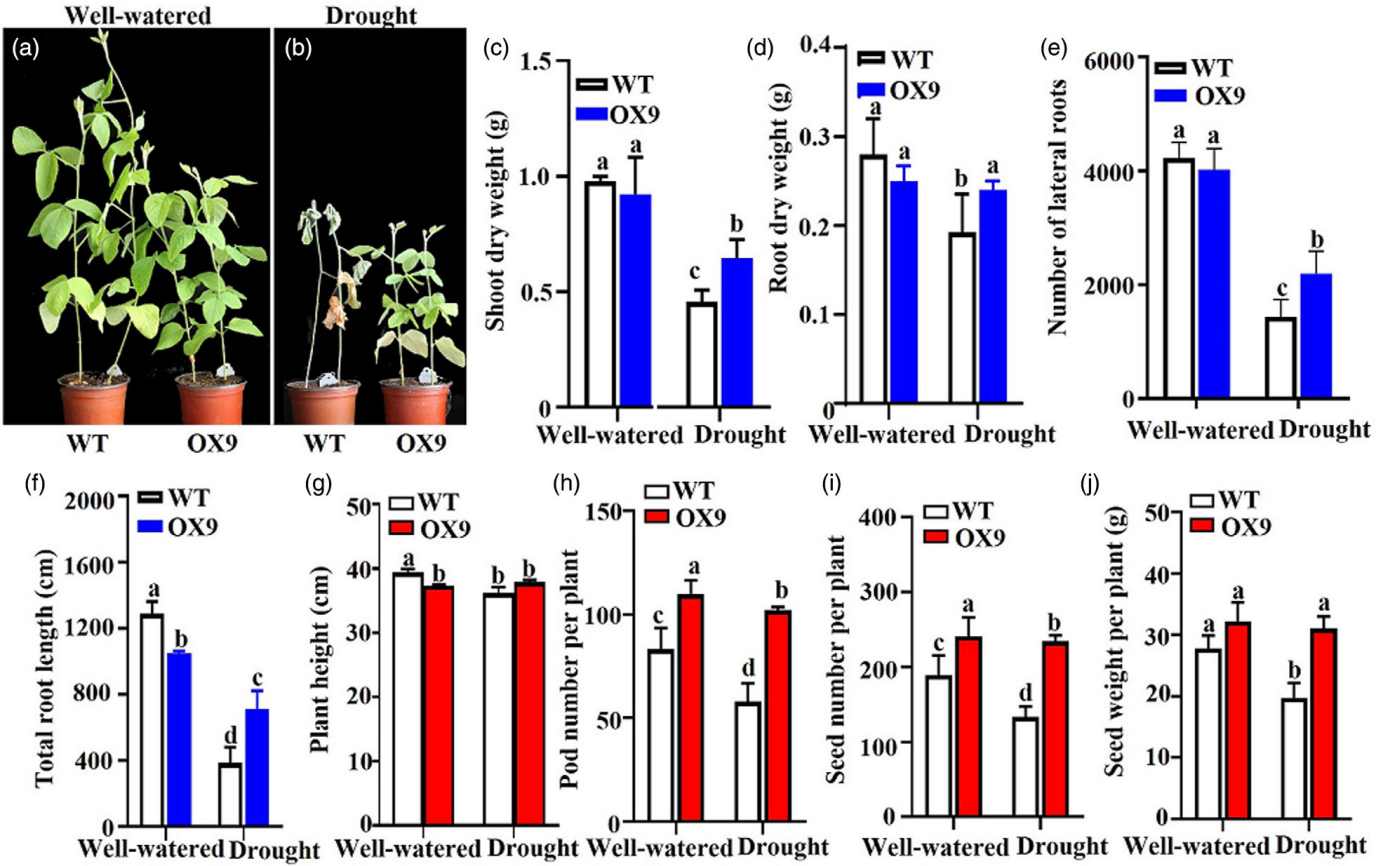
Plant architecture of OX9 transgenic and wild‐type (WT) plants under well‐watered and drought conditions. (a,b) Representative pictures showing plant architecture of 50‐day‐old homozygous T3‐generation OX9 and WT plants grown under well‐watered (a) and drought (b) conditions in a controlled growth room. Watering was withheld from 20‐day‐old soybean plants for 30 days. (c) Shoot dry weight, (d) root dry weight, (e) number of lateral roots and (f) total root length of 50‐day‐old homozygous T3‐generation OX9 and WT plants shown in (a,b). Data shown are means and standard deviations (*n* = 3 replicates; 5 plants/genotype/replicate). (g) Plant height, (h) pod number per plant, (i) seed number per plant and (j) seed weight per plant of T3‐generation OX9 and WT plants grown under well‐watered and drought conditions in the field in 2018. Data shown are means and standard deviations (*n* = 3 replicates; 5 plants/genotype/replicate). Significant differences were shown by different letters following Duncan’s multiple‐range test (*P* < 0.05).

Previously, Chen *et al*. (2013) reported that the *GmMYB14* was induced by dehydration, suggesting that it might have a role in soybean response to water‐deficit stress (Chen *et al*., 2013a). To verify this hypothesis, we first tested the responses of OX1 and OX9 plants to a polyethylene glycol (PEG) treatment and performed a survival test in soil pot under greenhouse conditions. The homozygous T2‐generation OX1 and OX9 seedlings showed a lesser wilting phenotype than WT in response to the presence of PEG (Figure S4a). Furthermore, when the plants were grown in soil and exposed to drought for 23 days, T2‐generation OX1 and OX9 plants displayed a lesser wilting and etiolated phenotype, consequently higher survival rates than WT after recovery (Figure S4b,c). Next, we selected homozygous T3‐generation OX9 as a representative line for a long‐term soil‐drying (30 days of drying) experiment under greenhouse conditions to assess various growth‐related parameters. Figure 5a–e showed that although OX9 plants showed a semi‐dwarf phenotype, no significant differences in shoot dry weight, root dry weight and number of lateral roots were observed between OX9 and WT plants grown under the well‐watered growth conditions. When soybean seedlings were exposed to a drought treatment, similar to previous observation, the OX9 plants displayed a lesser wilting and etiolated phenotype as compared with WT plants that clearly showed curled and dead leaves (Figure 5b). Furthermore, the OX9 plants exhibited lower decreases in shoot dry weight, root dry weight, number of lateral roots and total root length than WT plants under drought stress (Figure 5a–f). These results together provided a clear evidence that the overexpression of *GmMYB14* enhanced drought tolerance of transgenic soybean plants in the pot test under greenhouse conditions.

To further investigate whether the overexpression of *GmMYB14* could enhance drought tolerance and increase soybean yield under field conditions, we measured the effects of drought on yield‐related traits of the homozygous T3‐generation OX9 plants grown under field conditions at the 10‐cm plant density in HTBS in 2018. OX9 plants exhibited comparable PH, while WT plants showed a decrease in PH under drought in comparison with well‐watered conditions (Figure 5g). Importantly, the OX9 plants produced higher PN, SN and SW than did the WT plants under the limited water conditions of the field test (Figure 5h–j). The results of this field study convincingly demonstrated that the overexpression of *GmMYB14* could improve yield potential under water scarcity.

### Differentially expressed genes in *GmMYB14‐OX* and WT plants

**Figure 6:**
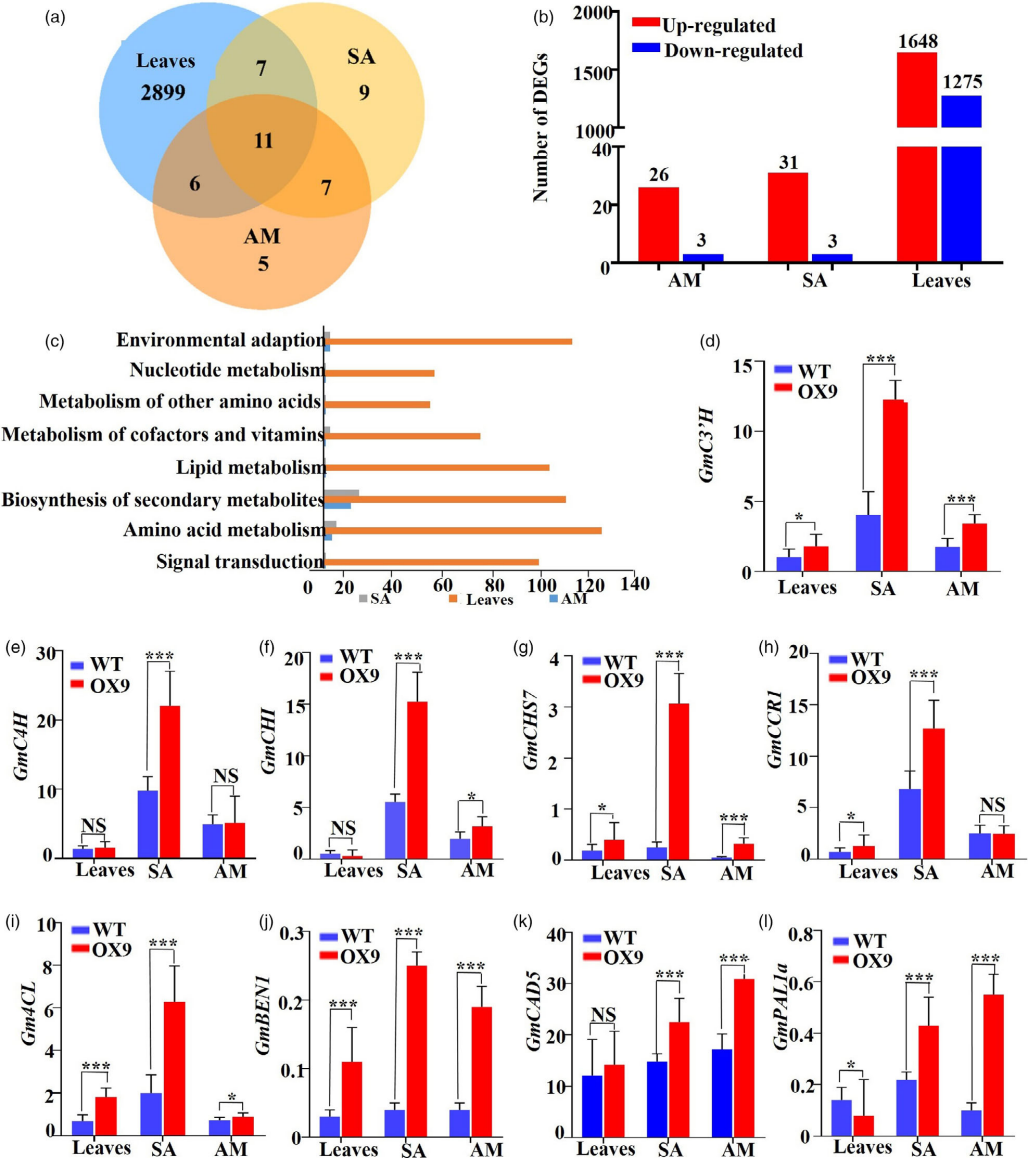
The classification of differentially expressed genes (DEGs) in OX9 versus wild‐type (WT) plants. (a) Venn diagram analysis of DEGs in the leaf, shoot apex (SA) and axillary meristem (AM) tissues of WT and OX9 plants. (b) The number of DEGs in the leaf, SA and AM tissues of WT and OX9 plants. (c) Pathway terms showing statistically significant enrichment in the number of DEGs in the leaf, SA and AM tissues of WT and OX9 plants. (d–l) Validation of the expression levels of 9 DEGs by qRT‐PCR. Relative expression levels were normalized to the expression level of *GmActin*. Statistically significant differences between OX9 (in leaves, SA or AM) and WT (in leaves, SA or AM) plants are marked with asterisks (*P < 0.05, **P < 0.01 and ***P < 0.001; Student's *t*‐test. NS, non‐significant).

To identify *GmMYB14*‐mediated genetic pathways underlying plant architecture, we investigated differentially expressed genes (DEGs) in soybean in response to the overexpression of *GmMYB14* gene by RNA‐seq using the axillary meristems (AM), shoot apex (SA) and leaf tissues collected from the WT and homozygous T3‐generation OX9 plants at day 24 after sowing (24 DAS). The results indicated that 29 (26 up‐regulated and 3 down‐regulated), 34 (31 up‐regulated and 3 down‐regulated) and 2923 (1648 up‐regulated and 1275 down‐regulated) DEGs, with at least twofold change in gene expression (*q*‐values ≤ 0.05), were identified in the AM, SA and leaf tissues, respectively, of the OX9 plants in comparison with the corresponding tissues of WT plants (Figure 6a,b and Tables S1–S3). These DEGs revealed statistically significant enrichment in up‐regulated genes for the pathways related to biosynthesis of secondary metabolites in all three AM, SA and leaf tissues (Figure 6c). Up‐regulated genes related to flavonoid, isoflavonoid and lignin biosyntheses, including the *G. max CHALCONE SYNTHASE* (*GmCHS7*/*Glyma.01G228700*), *G. max COMARATE 3‐HYDROXYLASE* (*GmC3’H*/*Glyma.03G122000*) and several members of the *G. max PHENYLALANINE AMMONIA‐LYASE* (*GmPAL*) family‐like *GmPAL1a*/*Glyma.03G181600*, *GmPAL2a*/*Glyma.10G058200* and *GmPAL2b*/*Glyma.13G145000*, were found in all three tissues (Tables S1‐S3). It was important to note that the *Glyma.18G220600*, which encodes a BRASSINOSTEROID‐INSENSITIVE 1 ENHANCED 1 (BEN1) homolog involved in BR catabolism based on the evidence that increased the expression of the *AtBEN1* gene in *Arabidopsi*s resulted in reduced endogenous BR levels and growth retardation (Yuan *et al*., 2007), was up‐regulated in the three tissues (Tables S1‐S3). In addition, several auxin‐related genes, including *G. max AUXIN RESPONSE FACTORS* (*GmARF*s/*Glyma.14G217700* and *Glyma.10G210600*), *G. max AUXIN‐BINDING PROTEINS* (*GmABP*s/*Glyma.02G150100* and *Glyma.15G176900*), *G. max AUXIN‐RESISTANT* (*GmAXR4*/*Glyma.03G237400*), *G. max 3‐INDOLEACETIC ACID* (*GmIAA1*/*Glyma.02G000500*) and *G. max LIKE AUXIN‐RESISTANT1* (*GmLAX1*/*Glyma.02G255800*), were up‐regulated in the leaves of OX9 versus WT plants (Table S3). We then performed a qRT‐PCR assay of 9 genes in the collected AM, SA and leaf samples to compare their expression changes detected by RNA‐seq and qRT‐PCR, which revealed consistent results between the two methods (Figure 6d–l and Tables S1–S3).

### GmMYB14 acts as a transcriptional activator of *GmBEN1* to potentially block BR effects

**Figure 7:**
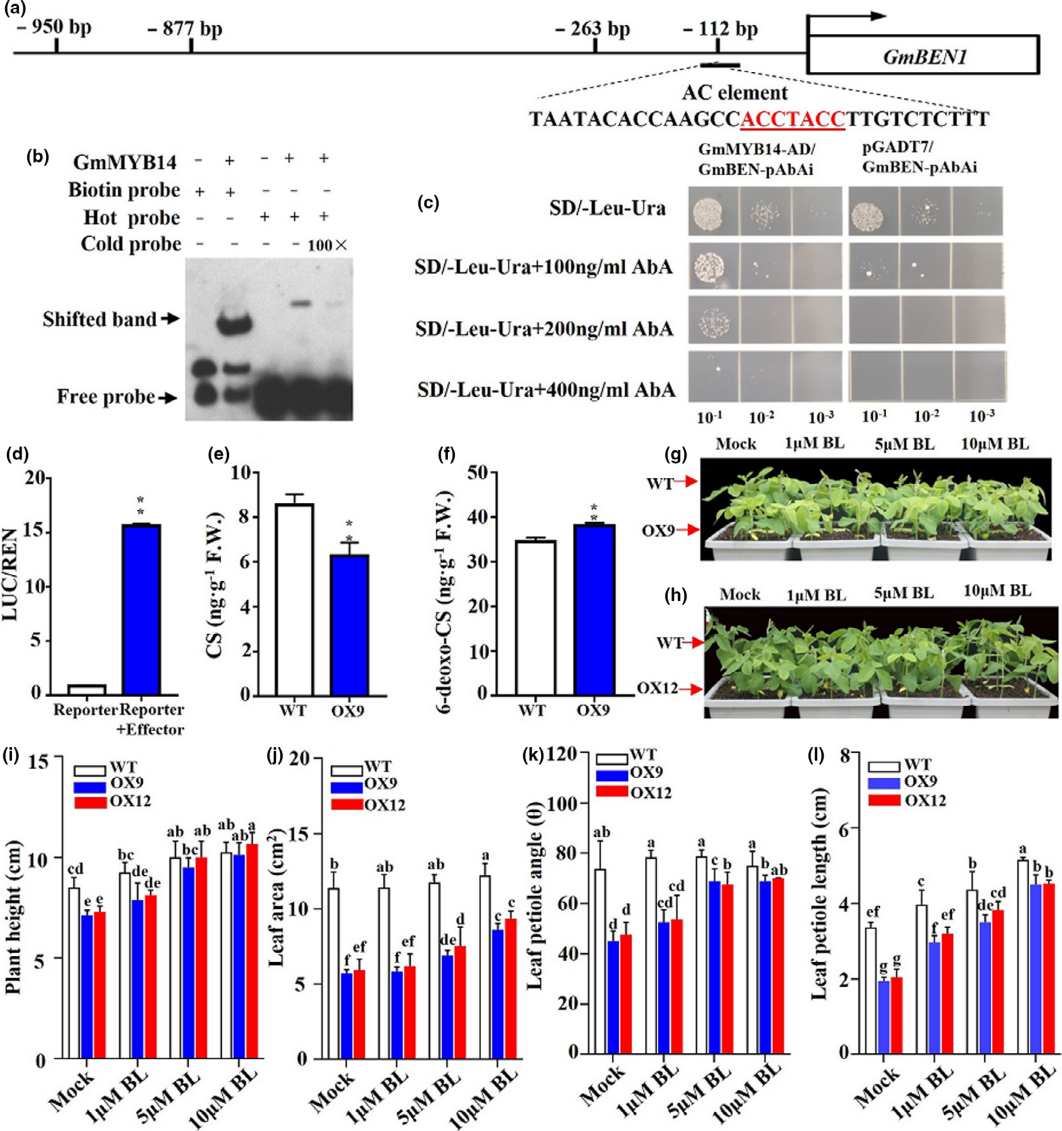
GmMYB14 directly activates the expression of *GmBEN1* gene. (a) Schematic diagram of the *GmBEN1* promoter with four putative AC elements ‘ACC(A/T)A(A/C)C’ within 1‐kb region upstream of the ATG. (b) Electrophoretic mobility shift assay (EMSA) indicates that the GmMYB14 binds to the first putative AC element ‘ACCTACC’ of the *GmBEN1* promoter shown in (a). The 30‐bp fragment containing the first AC element was used as a hot probe in the EMSA. Binding competition was tested using the 100× competitive cold probe. Biotin probe was used as negative control. (c) Yeast one‐hybrid assay indicated the binding of GmMYB14 to the 31‐bp sequence of the *GmBEN1* promoter. Yeast cells from serial dilutions (1:10, 1:100 and 1:1000) were grown on SD/‐Leu/‐Ura medium supplemented with different concentrations of aureobasidin A (AbA). The empty pGADT7 vector was used as negative control. (d) GmMYB14 protein promotes the transcription of *LUC* (*LUCIFERASE*) reporter gene driven by the 1‐kb pGmBEN1 promoter in*Arabidopsis*protoplasts. LUC activities were normalized to the respective *Renilla* luciferase (REN) activity and were expressed in relative expression units. Data shown are means and standard deviations of three replicates (*n* = 3), with asterisks showing statistically significant difference (**P < 0.01; Student's *t*‐test). (e–f) Contents of endogenous brassinosteroids, including (e) castasterone (CS) and (f) 6‐deoxo‐castasterone (6‐deoxo‐CS), in the shoots of 2‐week‐old OX9 transgenic and wild‐type (WT) plants. Data shown are means and standard deviations of three replicates (*n* = 3), with asterisks showing statistically significant differences (**P < 0.01; Student's *t*‐test). (g‐h) Phenotypes of 17‐day‐old WT plants, T3‐generation OX9 (g) and OX12 (h) after spaying with 0, 1, 5 or 10 μM brassinolide (BL). (i–l) Plant height, leaf area, leaf petiole angle and leaf petiole length of the OX9, OX12 and WT plants shown in (g–h). Data shown are means and standard deviations (*n* = 18 plants/genotype/treatment). Significant differences were shown by different letters following Duncan’s multiple‐range test (*P* < 0.05).

We hypothesized that the GmMYB14‐mediated up‐regulation of *Glyma.18G220600*/*GmBEN1* (Figure 6j and Tables S1–S3) might contribute to the semi‐dwarf phenotype of the OX9 plants (Figure 2a–b) by affecting the BR biosynthesis. To test this hypothesis, we investigated whether GmMYB14 could directly regulate the expression of *GmBEN1* gene in soybean. A previous study showed that AtMYB15 could bind to the AC elements [ACC(A/T)A(A/C)C] in *Arabidopsis* genome (Chezem *et al*., 2017). We analysed the promoter region of *GmBEN1* and identified four putative AC elements within the 1‐kb promoter region upstream of the ATG (Figure 7a). We, next, employed electrophoretic mobility shift assay (EMSA) using the 30‐bp fragment containing the first AC element (−119 to −112) as a probe DNA (Figure 7a), as well as transactivation assay in *Arabidopsis* protoplasts using the pGmBEN1*::LUC* reporter construct to validate whether GmMYB14 directly binds to the promoter region of *GmBEN1* and modulate its expression. Moreover, GmMYB14 specifically bound to the promoter of *GmBEN1* containing the AC element in yeast as shown by a yeast one‐hybrid assay (Figure 7c). Results of these experiments demonstrated that the GmMYB14 could bind to the identified AC element(s) in the *GmBEN1* promoter (Figure 7b,c) and activate the expression of *GmBEN1* gene (Figure 7d).

Previously, Yuan *et al*. (2007) reported that *Arabidopsis* mutant plants with increased expression of *AtBEN1* showed semi‐dwarf phenotype with rounded leaves by decreasing the endogenous BR levels (Yuan *et al*., 2007). To test whether *GmMYB14* regulates plant architecture through reducing the levels of endogenous BRs by directly up‐regulating the *GmBEN1* expression, we compared the contents of BRs, including BL, castasterone (CS) and 6‐deoxo‐castasterone (6‐deoxo‐CS), in the shoots of WT and OX9 plants. Results revealed that the CS content highly decreased, while the 6‐deoxo‐CS content slightly increased in the OX9 in comparison with WT plants (Figure 7e,f). The levels of BL were too low to be detected in both the OX9 and WT plants.

Next, we tested whether spraying exogenous BL to OX9 and OX12 plants could rescue their semi‐dwarf phenotype, which might provide a direct evidence for the semi‐dwarfism associated with the GmMYB14‐mediated decrease in BR levels in transgenic soybean plants. Thus, OX9, OX12 and WT plants were grown under controlled growth conditions, and 10‐day‐old seedlings were subsequently sprayed with 1, 5 and 10 μm BL or mock control. One week after the BL treatment, the plant height, leaf area, leaf petiole angle and leaf petiole length were measured. Results revealed that exogenous application of 5–10 μm BL rescued the semi‐dwarf phenotype of *GmMYB14‐OX* plants (OX9 and OX12) to the WT level (Figure 7g–i), and partially recovered their leaf area, leaf petiole angle and leaf petiole length (Figure 7j–l). Recently, Ye *et al*. (2017) reported that BR signalling pathway inhibits drought responses in plants. Figure S5a,b showed that the expression of *GmMYB14* and *GmBEN1* genes was also increased in the leaves of OX9 versus WT plants under PEG treatment. Taken together, these results indicated that the stress‐modulated GmMYB14 directly bound to the promoter of *GmBEN1* and up‐regulated its expression to reduce the endogenous BR levels, which contributed to regulating plant architecture and drought tolerance in soybean.

## Discussion

In the current study, we found that *GmMYB14* has a role in regulating plant architecture, including PH, leaf area, leaf petiole length, leaf petiole angle and primary root length (Figures 2, 3, 4 and Figures S1c, S2–S3), and in improving yield of transgenic soybean plants under field conditions, particularly when they were grown in high density within a row (Figure 4 and Figure S3). It is well‐known that BRs regulate plant architecture, including PH and lamina joint, and have a great role in crop improvement (Liu *et al*., 2019; Ren *et al*., 2019; Tian *et al*., 2019; Tong and Chu, 2018). In *Arabidopsis*, the AtBEN1 was reported to be involved in BR catabolism, perhaps as a steroid reductase, because the *ben1‐1D* gain‐of‐function mutant with increased expression of *AtBEN1* showed a decrease in endogenous BR levels and growth retardation (Yuan *et al*., 2007). We found that the overexpression of *GmMYB14* up‐regulated the expression of *GmBEN1* (Figure 6j and Tables S1‐S3), which was resulted from the direct activation of *GmBEN1* by GmMYB14 as evidenced by the results of EMSA, yeast one‐hybrid assay and transactivation assay (Figure 7b–d). Yuan *et al*. (2007) also reported that AtBEN1 might have a possible role in catalysing the conversion of CS, typhasterol (TL) and BL to the biologically inactive 6‐deoxo‐CS, 6‐deoxo‐TL and 6‐deoxo‐BL, respectively (Yuan *et al*., 2007). In the current study, we recorded that the endogenous CS level highly decreased but the 6‐deoxo‐CS level slightly increased in *GmMYB14*‐*OX9* transgenic line (Figure 7e,f), and the semi‐dwarf phenotype of *GmMYB14*‐*OX* plants (OX9 and OX12) was rescued by BL application (Figure 7g–i). These results suggest the involvement of *GmMYB14* in the catabolic pathway of BRs, particularly CS, through modulation of its downstream gene *GmBEN1*, which probably leads to reduction in total endogenous biologically active BR content, and consequently growth retardation of transgenic soybean plants overexpressing *GmMYB14* due to reduced cell growth rate (Figure 3). The observed reduced cell sizes of *GmMYB14*‐*OX* plants might be attributed to down‐regulation of *Glyma.14G203900* and *Glyma.02G235900* genes encoding cell wall‐modifying expansins (Figure 3m,n) (Yin *et al*., 2019). Genes encoding cell wall‐modifying enzymes such as extensins, expansins and xyloglucan endotransglucosylases are well‐known to be positively regulated by BRs (Yin *et al*., 2019).

Interestingly, our results revealed that the supplementation of exogenous BL could not fully restore the phenotype of *GmMYB14*‐overexpressing plants (Figure 7j–l), suggesting that the dwarfism resulted from *GmMYB14* overexpression might be associated with other pathway(s) in addition to the BR pathway. Recently, Liu *et al*. (2019) reported that the wheat TaSPL8 modulated leaf angle through both auxin and BR signalling pathways (Liu *et al*., 2019). Our RNA‐seq analysis also revealed that several auxin‐related genes, including *GmARF*s, *GmABP*s, *GmAXR4*, *GmIAA1* and *GmLAX1*, were up‐regulated in the leaves of OX9 versus WT plants (Table S3), indicating that *GmMYB14* regulates plant architecture, perhaps partially through the auxin pathway. It will be interesting to precisely establish the regulatory network of the GmMYB14‐mediated auxin signalling involved in the regulation of soybean plant architecture and drought tolerance, by proving the *in planta* functions of these identified candidates, in future studies.

It should be mentioned that the present research also showed that in comparison with WT plants, *GmMYB14‐OX* plants displayed semi‐dwarf (OX1, OX9 and OX12) architecture at the V5 and V6 vegetative stages when they were grown in greenhouse and field conditions (Figure 2a,b,f and Figure S1c). However, there were no remarkable differences in PH between *GmMYB14‐OX* and WT plants at reproduction (R1) (Figure 2g) and harvest stages (Figure 4a,b and Figure S3a,i–j), when they were cultivated under field conditions. This observation might be explained by the fact that *GmMYB14‐OX* plants had more nodes and more branches than WT plants at the harvest stage (Figure 4d–e and Figure S3c,d, l–m). Further studies need to be conducted to investigate the differences in *GmMYB14‐OX* plants observed at vegetative and reproduction stages. In addition, to assess the function of *GmMYB14* in regulating plant architecture using a loss‐of‐function approach, a target adaptor in the first intron and second exon of *GmMYB14* gene was chosen for mutagenesis in soybean using the CRISPR/Cas9 system (Figure S6a). We obtained two T0 transgenic lines, and sequence analysis showed that these two lines both had an 8‐bp deletion in the target site in the second exon of *GmMYB14* gene (Figure S6b). However, there were no significant differences in plant architecture observed between WT and the *gmmyb14* mutant plants (Figure S6c), which might be due to functional redundancy of the GmMYB TFs grouped into the AtMYB14 clade (Figure 1a).

It has been reported that several TFs exert opposite effects on regulating BR responses and drought tolerance (Chen *et al*., 2017; Xie *et al*., 2019). Results of our RNA‐seq analysis revealed that several DEGs involved in the BR pathway might have a role in regulating plant architecture and drought tolerance (Figure S5b–h and Tables S1‐S3). The *RESPONSIVE TO DESICCATION 26* (*RD26*/*ANAC072*) gene, encoding a NAC (NO APICAL MERISTEM, *ARABIDOPSIS* TRANSCRIPTION ACTIVATION FACTOR1/2 AND CUP‐SHAPED COTYLEDON) protein that acts as a positive regulator of drought tolerance in *Arabidopsis* (Tran *et al*., 2007; Tran *et al*., 2004; Ye *et al*., 2017), interacts with the BR signalling member BRI1 EMS SUPPRESSOR 1 (BES1) to inhibit BES1 transcriptional activity on BR‐regulated genes, consequently the BR‐controlled phenotype (Ye *et al*., 2017). Interestingly, the BR signalling loss‐of‐function mutant *bri1‐5* plants exhibited increased drought tolerance in *Arabidopsis* through alleviating the negative effect of BR signalling on the expression of *RD26* and its homologs (Ye *et al*., 2017). In addition, the BKI1 negatively regulates BRI1 in BR pathway to regulate the plant architecture in *Arabidopsis* (Wang *et al*., 2007a,b). The overexpression of *UGT73C5* also showed BR‐deficient phenotype in *Arabidopsis* (Peppenberger et al., 2005). In agreement with this finding, eight genes including one *GmBEN1*/*Glyma.18G220600*, one *GmBKI1*/*Glyma.06G039100* and four *GmUGT73C5* genes (*Glyma.03G186900*, *Glyma.03G187000*, *Glyma.19G187000* and *Glyma.19G187100*) were up‐regulated, while one *GmBRI1*/*Glyma.06G147600* was down‐regulated in the leaves of OX9 plants, compared with WT, under PEG treatment (Figure S5b–h). We also examined the expression of several drought‐responsive marker genes and found that these representative genes were all induced by PEG treatment in PEG‐treated OX9 plants (Figure S5i–m). Taken together, our findings provide compelling evidence for the *GmMYB14‐*mediated repression of BR signalling, leading to enhanced drought tolerance of *GmMYB14‐OX* plants under both control growth and field conditions.

In conclusion, our studies showed that *GmMYB14* plays important functions in regulating plant architecture, yield and drought tolerance in soybean. Overexpression of *GmMYB14* stably altered the plant architecture by affecting cell growth and increased yields under field conditions particularly when they were grown in high density. Importantly, further comparative studies under field conditions with sufficient and deficient water supply revealed that the overexpression of *GmMYB14* in soybean improved drought tolerance and increased yield. Molecular studies revealed that GmMYB14 mediates at least BR signalling pathways to achieve ideal plant architecture and drought tolerance in soybean. Findings reported in this study provide a comfortable strategy for stably increasing yield and drought tolerance in soybean, as well as other crops, by altering the expression of a single gene encoding a MYB TF using the gain‐of‐function approach.

## Methods

### Plant materials and growth conditions

Soybean cultivar Tianlong No. 1 (WT) and two transgenic *GmMYB14‐OX* lines were planted in field at the HTBS of our institute (30°40′41″N and 113°43′39″E) for two years, in 2018 and 2019, using a randomized complete block design with 3 replications. In 2018, each plot contained five rows with a row length of 3 m, a distance of 40 cm between rows and a 10‐cm interval distance between two plants within a row. In 2019, each plot contained five rows with a row length of 3 m, a distance of 40 cm between rows, and three different types of plant density, specifically 5‐, 10‐ or 20‐cm interval distance between two plants within a row, were applied for testing the effect of plant density under field conditions. Plant architecture characteristics, including PH, NN and BN, and yield‐related traits, including PN, SN and SW in soybean, were measured at R1 reproductive and harvest stages, respectively.

For plant architecture analysis and gene expression analysis under the control growth room conditions, the WT, OX1, OX7, OX9, OX10 and OX12 plants were grown in pots (height × top diameter = 18.5 × 18.5 cm) with one plant per pot in an artificial climate chamber under 16‐h light/8‐h dark photoperiod conditions at 28°C. Plant architecture features, including PH, leaf area, leaf petiole length and leaf petiole angle of WT and *GmMYB14‐OX,* were measured at V5 vegetative stage. Roots, stems, leaves, leaf petioles and shoot apices were harvested from WT at V3 stage, flowers were harvested from WT at R2 stage, and pods and immature seeds were harvested from WT at R5 stage for RNA purification. The harvested samples were stored at –80°C until total RNA extraction was performed for gene expression analyses.

For the analysis of primary root length under hydroponic conditions, seeds of WT, OX1 and OX9 were germinated on water‐soaked papers for 5 days, and then, the seedlings were transferred into half‐strength Hoagland nutrient solution in a growth chamber. Primary root lengths were measured, when seedlings were at V2 vegetative stage. For the analysis of *GmMYB14* expression in the presence of different concentrations of exogenous BL, WT seedlings, which had the first trifoliolate leaves unfolded, were treated with half‐strength Hoagland nutrient solutions containing 0, 0.1, 1.0, 5.0 and 10 μm BL, respectively, for 3 h. For analysis of *GmMYB14* expression treated with BL for different time periods, WT seedlings were treated with half‐strength Hoagland nutrient solution containing 5 μm BL for 0, 3, 6 and 9 h. RNA was extracted from collected leaf samples for expression analysis using qRT‐PCR.

### Drought stress treatments

For drought stress treatments, transgenic soybean seedlings were grown in a growth chamber. Two types of drought treatments, namely an osmotic stress (PEG) treatment and a pot soil‐drying treatment, were employed in the present study to evaluate drought tolerance of transgenic plants. For PEG treatment, WT and homozygous T2‐generation seeds (OX1 and OX9) were germinated on water‐soaked papers for five days. Germinated seedlings (*n* = 15 plants/genotype/treatment) were transferred into the hole of foam board on a plastic box (length × width × height = 30 × 25 × 13 cm) filled with water. 10 days later, the seedlings were transferred into the solution with 15% PEG for 1 day and then with 25% PEG for 2 days. Treated WT and transgenic plants (OX1 and OX9) were investigated and photographed for the evaluation of the PEG effect on plant phenotype (Figure S4a).

For pot soil‐drying experiment I, WT, OX1 and OX9 were grown in the same pots (height × top outer diameter = 14.5 × 19 cm) containing a mixture of soil and vermiculite (2:1, 500 g/per pot) (*n* = 3 biological replicates; 6 plants/genotype/replicate, one plant/pot). The saturated soil water content (SWC) was about 40%. Water was withheld from day 25 after sowing (25 DAS), and SWC was measured on every alternate day. Approximately 18 days after water withholding, the SWC reached below 10%, seedlings showed wilted and etiolated symptoms, and plants were photographed (Figure S4b). 23 days after water withholding, rewatering was then conducted, and plant survival rates were estimated after 5 days of recovery (Figure S4c).

For pot experiment II, WT and homozygous T3‐generation OX9 plants were grown in separate pots (height × top outer diameter = 12.5 × 14 cm) with two plants per pot with mixture of soil and vermiculite (2:1, 280 g/per pot). The WT and *GmMYB14‐OX* plants were well‐watered, and the SWC was about 40% at 20 DAS. Thereafter, water was withheld for 30 days, and the plants were photographed for phenotype evaluation (Figure 5). Roots of transgenic and WT plants at V5 stage were also collected to investigate RSA in a box filled with water. The number of lateral roots as number of tips, including primary, secondary and tertiary laterals, and total root length (*n* = 12 plants/genotype/treatment) were analysed using the WinRHIZO software (Prince *et al*., 2019). Shoot dry weight and root dry weight of collected plants were also measured (*n* = 12 plants/genotype/treatment).

For drought stress treatments under field conditions, the WT and the homozygous T3‐generation OX9 plants were grown in field at the HTBS of our institute in three replications under well‐watered and water‐stressed conditions in 2018. In well‐watered field, the seedlings were irrigated normally, while in water‐stressed field, water was withheld for 52 days from the V2 stage (the first trifoliate was fully expanded) to the R1 stage (plants with first flower). PH, PN, SN and SW were measured after the harvest to evaluate the effect of drought on plant growth and yield parameters.

### Gene expression analyses

Total RNA was extracted from WT and transgenic soybean lines (OX1, OX7, OX9 and OX12) using RNeasy Plant Mini Kit (Takara, Dalian, China). About 1 µg of total RNA was used for reverse transcription using HiScript 1st Strand cDNA Synthesis Kit (Vazyme, Nanjing, China). qRT‐PCR procedures were performed as previously described (Chen *et al*., 2013a). The reference gene used was the soybean *GmActin* gene, and the reactions were run on the CFX‐Connect™ Real‐Time PCR System (Bio‐Rad, California, USA) using the iTaq™ Universal SYBR Green Supermix. Primers used in qRT‐PCR are shown in Table S4. Data from three biological replicates were analysed following the relative quantification method (2^−ΔCT^).

### Gene constructs and plant transformation

*Glyma19g164600*/*GmMYB14* (GenBank Accession Number KC979136) was found to display differential expression patterns in drought‐tolerant cultivar Jindou21 and drought‐sensitive cultivar Zhongdou33 (Chen *et al*., 2013a). The full‐length CDS of *GmMYB14* was amplified by PCR from a soybean cultivar Tianlong No. 1 cDNA library, which was identical to the CDS cloned from cultivars Jindou21 and Zhongdou33, and was subsequently cloned into the pB2GW7 expression vector (Figure S1a). Construction of the pYLCRISPR/Cas9P35S‐*gmmyb14* plasmid was performed following the method described by Bao *et al*. (2019) with some minor modifications. In brief, the target adaptor was designed using the web tool CRISPR‐P (http://cbi.hzau.edu.cn/crispr/), integrated into the sgRNA expression cassettes driven by the *A. thaliana* U3 promoter and then was built into the pYLCRISPR/Cas9P35S‐BS vector. The resulting constructs were verified by sequencing and were then transferred into the *Agrobacterium tumefaciens* EHA105. EHA105 recombinant strain, carrying the p35S*::GmMYB14* plasmid or pYLCRISPR/Cas9P35S‐*gmmyb14* plasmid, was used to transform Tianlong No. 1 cultivar according to the procedure previously described (Bao *et al*., 2019).

### Identification of transformants

Soybean transformants were identified by southern blot as described by Cao *et al*. (2011). Briefly, approximately 40 μg of DNA from each sample was cut with HindIII overnight. DNA fragments were separated by electrophoresis on 0.8% agarose gel and were then transferred to a Hybond‐N + nylon membrane (Amersham) and cross‐linked by baking at 80°C for 2 h. DIG‐dUTP‐labelled PCR product of *Bar* gene was applied as a probe for hybridization, which was performed following the manufacturer’s instruction (DIG‐High Prime DNA Labeling and Detection Starter Kit II, Roche, Basel, Switzerland).

### Transcriptome analysis using RNA‐seq

The axillary meristem, shoot apex and leaf tissues samples of WT and OX9 plants were collected from the seedlings at V2 vegetative stage (at day 24 after sowing). Each sample collected from 10 individual plants, and three biological replicates were analysed. Total RNA was extracted from each sample, and then, the cDNA was synthesized with adapters and sequenced with the Illumina HiSeq 2500 analyzer at BGI Technologies (Shenzhen, China).

### Subcellular localization of GmMYB14‐GFP fusion proteins

The full‐length CDS of *GmMYB14* was amplified with two primers GmMYB14‐SL‐F/R (Table S4). The PCR product was subcloned into the pJG053 vector under the control of CaMV *35S* promoter. The fusion construct was introduced into lower epidermal cells of tobacco leaves using *A. tumefaciens* GV3101 strain as previously described (Sparkes *et al*., 2006). GFP alone was used as the control, and FIBRILLARIN2‐mCherry (FIB2‐mCherry) was used as a nucleolar marker (Missbach *et al*., 2013). Fluorescence of the transformed leaves was assessed 72 h post‐infection using a Nikon A1 confocal microscopy (Nikon, Tokyo, Japan). The excitation laser wavelengths are 488 nm for GFP and 570‐620 nm for FIB2‐mCherry.

### Transactivation activity assay

The *GmMYB14* full‐length CDS was cloned into the pGBKT7 plasmid to assess protein transactivation activity in the yeast strain AH109. Primers used for PCR amplification are listed in Table S4. The empty pGBKT7 plasmid was used as a negative control. All transformed cells were selected on SD medium plates without tryptophan (e.g. SD/‐Trp). The cell concentrations of yeast transformants were adjusted to an OD_600_ of 0.01 to 1.0, and the yeast cells were drop‐inoculated onto the SD plates without tryptophan (SD/‐Trp), or SD plates containing X‐α‐Gal but lacking tryptophan, histidine and adenine (e.g. SD/‐Trp/‐His/‐Ade + X‐α‐Gal). After incubating the plates at 30°C for 3 days, photographs were taken.

### Dual‐luciferase assay

The 1000‐bp DNA sequence upstream of the ATG start codon of *GmBEN1*/*Glyma.18G220600* gene was PCR‐amplified from cultivar Tianlong No. 1 and was then subcloned into vector pGreenII‐LUC to generate pGmBEN1::LUC reporter plasmid. Vector pGreenII‐LUC without promoter insertion was used as a control. The effector plasmid was constructed by subcloning the *GmMYB14* full‐length CDS into the vector pAN580 under the control of the *35S* promoter. The reporter plasmid and control plasmid were either co‐transformed with the effector plasmid or transformed alone into *Arabidopsis* protoplasts as described previously (Yoo *et al*., 2007). The dual‐luciferase reporter assay system E1910 (Promega, Wisconsin) was used to determine the luminescence activity in each sample. Primers used for transient expression assay are listed in Table S4.

### Electrophoretic mobility shift assay

EMSA was performed basically following the previous method described by Nan *et al*. (2014). Briefly, the full‐length CDS of *GmMYB14* was amplified by PCR and then cloned into the NdeI/XbaI sites of the expression vector pCzn1‐His using the ClonExpress II One Step Cloning Kit (Vazyme, Nanjing, China) to express the GmMYB14 protein in *Escherichia coli* DE3 strain. The recombinant GmMYB14 protein was purified using the Ni affinity chromatography. The DNA products of the *GmBEN1* promoters were produced by annealing the forward and reverse complementary oligos containing the AC element ‘ACCTACC’. EMSA was performed using the EMSA Kit (Thermo Fisher Scientific, Shanghai, China). DNA–protein complexes were separated on a non‐denaturing 6% polyacrylamide gel, transferred to a positive nylon membrane and UV cross‐linked. Detection of the DNA–protein complex was probed with streptavidin‐HRP conjugate and incubated with the substrates of the enhanced chemiluminescence (ECL) kit (Amersham, Buckinghamshire, UK).

### Yeast one‐hybrid assay

To test the interaction between *GmMYB14* and the corresponding *cis‐*element of *GmBEN1*, the full‐length CDS of *GmMYB14* was amplified by PCR and inserted into pGADT7 (Clontech, Dalian, China) to build the effector vector (GmMYB14‐AD). The DNA fragment of the *GmBEN1* promoter was produced by annealing the forward and reverse complementary oligos containing the AC element ‘ACCTACC’, which was then cloned into the pAbAi vector to construct the bait (GmBEN‐pAbAi). The bait‐reporter yeast strain was generated by homologous integration of the bait into the Y1HGold genome (Clontech), and the effector vector was then transformed into the bait‐reporter strain for the determination of DNA–protein interaction. The transformed cells were cultured on SD/‐Leu/‐Ura medium supplemented with aureobasidin A (AbA) and incubated for 3 days at 30°C. Subsequently, yeast cells with different dilutions (1:10, 1:100 and 1:1000) were dropped on SD/‐Leu/‐Ura medium with different concentrations of AbA. The pGADT7 empty vector was used as negative control. The primers used in this assay are listed in Table S4.

### Analysis of endogenous BR contents

Soybean plants were grown in pots (height × top diameter = 18.5 × 18.5 cm) with two plants per pot in an artificial climate chamber under 16‐h light/8‐h dark photoperiod conditions at 28°C. Shoot parts were harvested from two‐week‐old seedlings. Three biological replicates per line were used in analysis. BR contents were quantified using high‐performance liquid chromatography‐electrospray ionization‐tandem mass spectrometry (HPLC‐ESI‐MS/MS) as previously described by Wang *et al*. (2015).

### Complementary assay by exogenous BL treatment

The BL treatment was performed following the methods reported by Yin *et al*. (2019). Briefly, the BL was diluted in dimethyl sulfoxide (DMSO) to make solutions with 1, 5 or 10 μm final concentration, which were then used to spray the shoot parts of 10‐day‐old soybean seedlings grown in greenhouse. Mock control was prepared in DMSO in the same way. Seven days after spay, photographs were taken, and plant height, leaf area, leaf petiole angle and leaf petiole length were measured. The same experiment was repeated at least twice, and photographs were taken to show representative results.

### Histological analysis

Plant samples were prepared according to a published method (Zheng *et al*., 2019), with some modifications. Briefly, leaf petiole and stem segments from two‐week‐old OX9 and WT seedlings were fixed in the FAA (5 mL of 38% formalin, 5 mL of 100% glacial acetic acid and 90 mL of 70% alcohol) solution overnight, followed by a series of dehydration and infiltration steps. Subsequently, the samples were embedded in paraffin. The tissues were sliced to receive a thickness of 8–10 µm (Leica RM2265) and were then stained with safranin and fast green. Finally, these prepared samples were observed using an Eclipse E80i light microscope (Nikon, Tokyo, Japan).

### Statistical analysis

If not specified, experiments were conducted with three replicates. The values were the means ± standard deviations (SDs). Data were subjected to Duncan’s multiple‐range test to determinate significant differences.

### Accession Numbers

RNA sequencing data are available at the NCBI Sequence Read Archive (http://www.ncbi.nlm.nih.gov/sra) under the accession numbers SAMN14614943, SAMN14614944, SAMN14614945, SRR10150567, SRR10150566, SRR10150565, SAMN14614946, SAMN14614947, SAMN14614948, SAMN14614949, SAMN14614950, SAMN14614951, SAMN14614952, SAMN14614953, SAMN14614954, SAMN14614955, SAMN14614956 and SAMN14614957.

## Conflicts of interest

The authors declare that they have no competing interests.

## Author contributions

L. Chen, L.‐S.P. Tran, X. Zhou and D. Cao conceived and designed the experiments; L. Chen, H. Yang and Y. Fang constructed the vectors and performed the field experiments and qRT‐PCR analysis; H. Chen and W. Guo performed EMSA and investigation of phenotypic parameters; H. Yang performed dual‐luciferase assay; L. Chen, X. Zhang and W. Dai performed the *Agrobacterium*‐mediated transformation and generated the transgenic soybean plants; S. Chen, Q. Hao and X. Liu determined the BR contents; D. Qiu, Z. Shan, Z. Yang and S. Yuan performed RNA‐seq analysis; C. Zhang performed the BL treatment; and L.‐S.P. Tran, L. Chen and D. Cao wrote the paper. All the authors read and finalized the version before submission.

## Supporting information


**Figure S1** Construction and analysis of the *GmMYB14‐OX* lines.
**Figure S2** The primary root lengths of the wild‐type (WT), OX1 and OX9 plants grown under hydroponic conditions.
**Figure S3** Plant architecture and yield component traits of wild‐type (WT) and *GmMYB14‐OX* plants grown under field conditions in 2018 and 2019.
**Figure S4** Tolerance assays of *GmMYB14‐OX* (OX1 and OX9) plants grown under the presence of polyethylene glycol (PEG), or drought conditions.
**Figure S5** Expression of brassinosteroid (BR)‐related genes and drought‐related marker genes in wild‐type (WT) and OX9 plants exposed to polyethylene glycol (PEG) treatment.
**Figure S6** CRISPR/Cas9‐mediated targeted mutagenesis of *GmMYB14* showed no significant differences in plant architecture of the mutated soybean plants.

**Table S1** Differentially expressed genes in axillary meristems of *GmMYB14‐OX* (OX9) versus wild‐type plants (WT), with fold changes ≥ 2.0 (up‐regulation indicated by red‐coloured letters) or ≤ −2.0 (down‐regulation indicated by blue‐coloured letters), and adjusted *P*‐values (*q*‐values) ≤ 0.05.
**Table S2** Differentially expressed genes in shoot apices of *GmMYB14‐OX* (OX9) versus wild‐type plants (WT), with fold changes ≥ 2.0 (up‐regulation indicated by red‐coloured letters) or ≤ −2.0 (down‐regulation indicated by blue‐coloured letters), and adjusted *P*‐values (*q*‐values) ≤ 0.05.
**Table S3** Differentially expressed genes in leaves of *GmMYB14‐OX* (OX9) versus wild‐type plants (WT), with fold changes ≥ 2.0 (up‐regulation indicated by red‐coloured letters) or ≤ −2.0 (down‐regulation indicated by blue‐coloured letters), and adjusted *P*‐values (*q*‐values) ≤ 0.05.
**Table S4** Primers used in plasmid constructions, southern blot analysis, qRT‐PCR analysis, electrophoretic mobility shift assay and transient expression assay.
