# Supplementary material for: Overexpression of GmMYB14 improves high‐density yield and drought tolerance of soybean through regulating plant architecture mediated by the brassinosteroid pathway
Source: Plant Biotechnol J. 2020 Nov 23;19(4):702–16. doi: 10.1111/pbi.13496 (PMC8051608; doi:10.1111/pbi.13496)
Supplement: Supplementary file 1 — Figure S1 Construction and analysis of the GmMYB14‐OX lines. Figure S2 The primary root lengths of the wild‐type (WT), OX1 and OX9 plants grown under hydroponic conditions. Figure S3 Plant architecture and yield component traits of wild‐type (WT) and GmMYB14‐OX plants grown under field conditions in 2018 and 2019. Figure S4 Tolerance assays of GmMYB14‐OX (OX1 and OX9) plants grown under the presence of polyethylene glycol (PEG), or drought conditions. Figure S5 Expression of brassinosteroid (BR)‐related genes and drought‐related marker genes in wild‐type (WT) and OX9 plants exposed to polyethylene glycol (PEG) treatment. Figure S6 CRISPR/Cas9‐mediated targeted mutagenesis of GmMYB14 showed no significant differences in plant architecture of the mutated soybean plants. [file PBI-19-702-s001.docx]

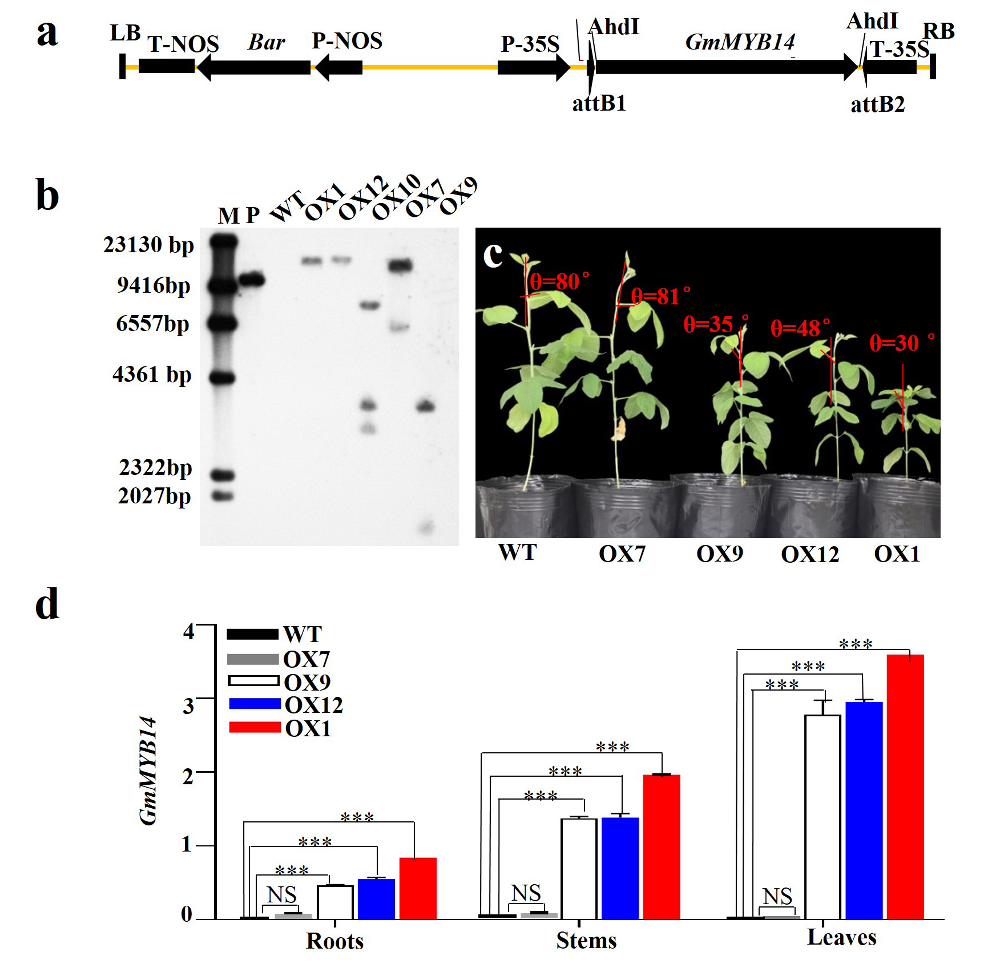


**Figure S1. Construction and analysis of the *GmMYB14-OX* lines.** (a) Diagram of p35S:*GmMYB14* plasmid*.* (b) Southern blot analysis of OX1, OX7, OX9, OX10 and OX12 lines. *Bar* gene was used as the probe in hybridization with HindIII-digested genomic DNA isolated from the transgenic soybean lines. M, DIG-DNA molecular marker; P, plasmid (positive control); WT, wild-type plants (negative control). (c) Representative pictures showing plant architecture of WT and T2-generation transgenic plants grown under the controlled growth conditions (16-h light/8-h dark photoperiod, 28°C) at day 30^th^ after sowing. The red numbers and lines indicated leaf angles (WT, Ɵ = 80º; OX7, Ɵ =81º; OX9, Ɵ = 35º; OX12, Ɵ = 48º; OX1, Ɵ = 30º). (d) Expression levels of *GmMYB14* gene in roots, stems and leaves of WT and *GmMYB14-OX* at the V5 vegetative stage. The plants were grown in an artificial climate chamber under 16-h light/8-h dark photoperiod conditions at 28°C. Data shown are means and standard deviations (*n* = 3 biological repeats/genotype). Statistically significant differences between each transgenic line and WT are marked with asterisks (****P* < 0.001; NS, nonsignificant, Student’s *t*-test).


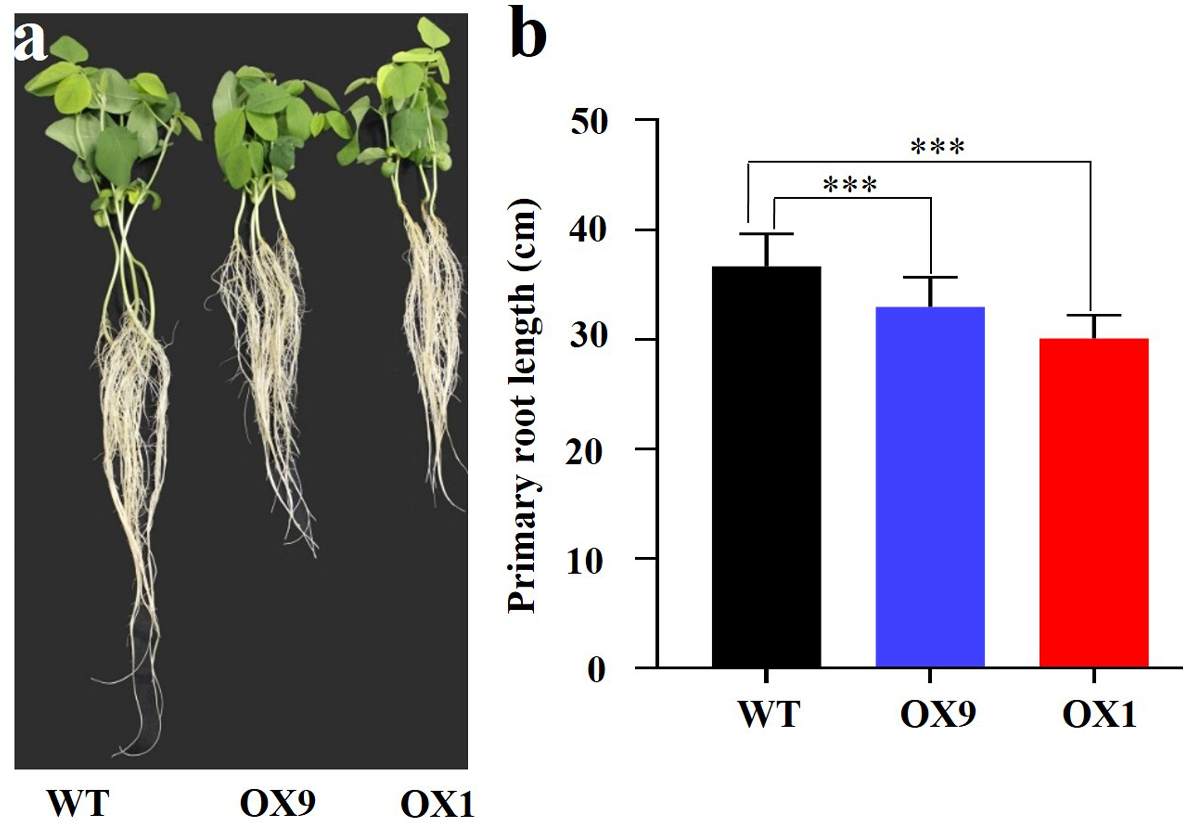


**Figure S2. The primary root lengths of the wild-type (WT), OX1 and OX9 plants grown under hydroponic conditions.** (a) Representative pictures of primary root lengths of WT, OX1 and OX9 plants grown under half-strength Hoagland nutrient solution in an artificial climate chamber (16-h light/8-h dark photoperiod and 28°C). The pictures were taken at the V2 vegetative stage. (b) Quantitative data of primary root lengths of WT, OX1 and OX9 plants at the V2 stage. Data shown are means and standard deviations of three experiments (*n* = 3; 12 plants/experiment), with asterisks showing statistically significant differences over the WT (****P* < 0.001; Student’s *t*-test).


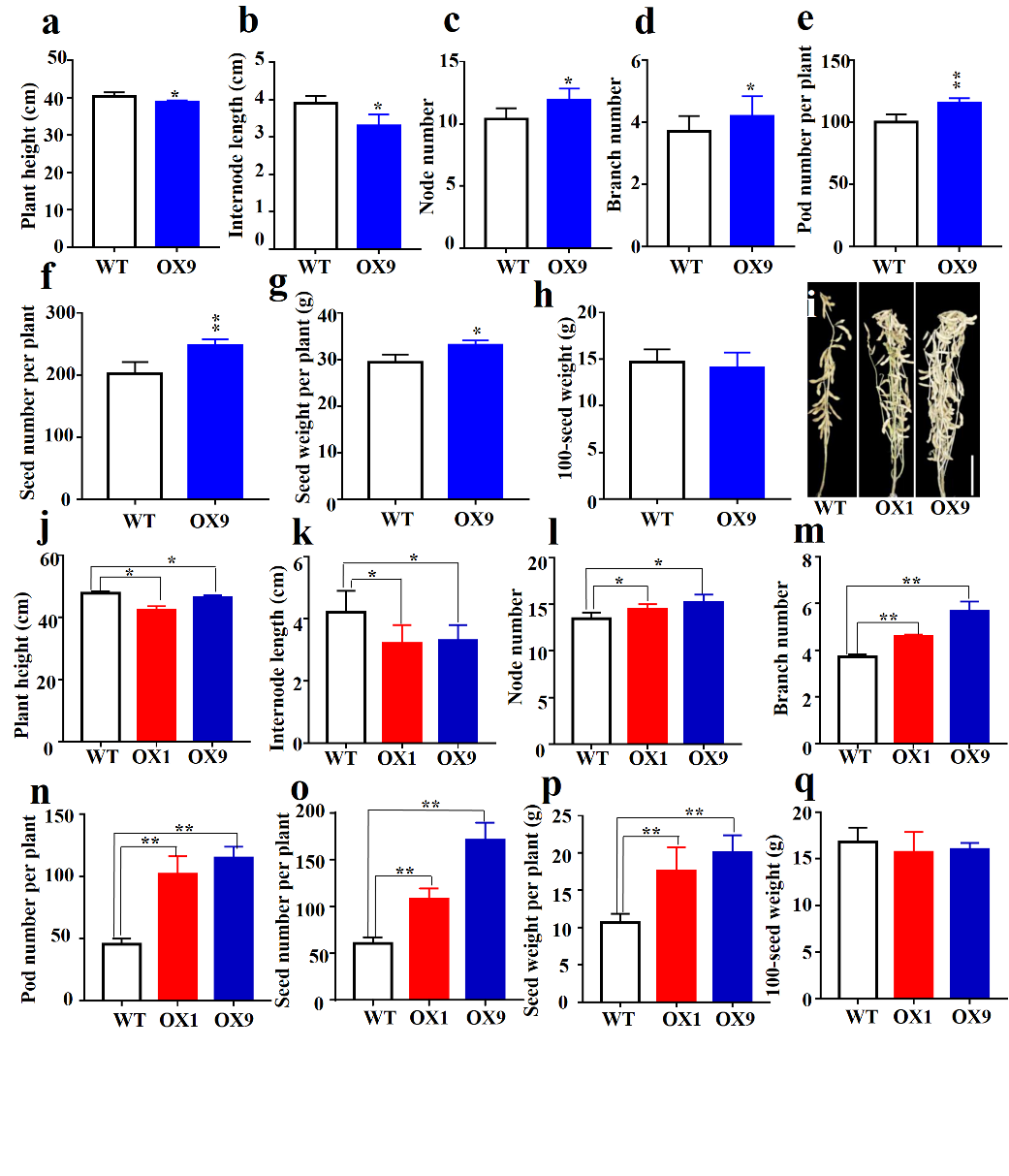


**Figure S3. Plant architecture and yield component traits of wild-type (WT) and *GmMYB14-OX* plants grown under field conditions** **in 2018 and 2019.** (a) Plant height, (b) internode length, (c) node number on main stem, (d) branch number on main stem, (e) pod number per plant, (f) seed number per plant, (g) seed weight per plant, and (h) 100-seed weight of WT and homozygous T3-generation OX9 plants grown under field conditions at 10-cm interval distance between two plants within a row in 2018. (i) Representative pictures showing plant architecture (bar = 10 cm), (j) plant height, (k) internode length, (l) node number on main stem, (m) branch number on main stem, (n) pod number per plant, (o) seed number per plant, (p) seed weight per plant, and (q) 100-seed weight of WT, homozygous T3-generation OX1 and T4-generation OX9 plants grown under field conditions at 10-cm interval distance between two plants within a row in 2019. Data shown are means and standard deviations of three replicates (*n* = 3 replicates; 5 plants/genotype/replicate). Statistically significant differences between each transgenic line and WT are marked with asterisk(s) (**P* < 0.05, ***P* < 0.01; Student’s *t*-test).


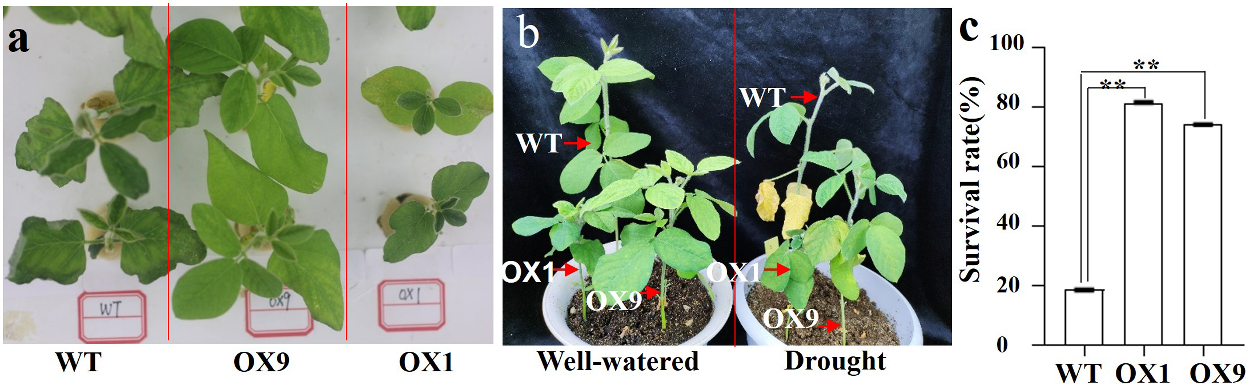


**Figure S4. Tolerance assays of *GmMYB14-OX* (OX1 and OX9) plants grown under the presence of polyethylene glycol (PEG), or drought conditions. (**a) Representative pictures showing the phenotype of wild-type plants (WT) and T2-generation transgenic plants (OX1 and OX9) exposed to PEG treatment. After germination in water-soaked papers for five days, transgenic and WT soybean seedlings were transferred into holes of foam board on a plastic box (length×width×height = 30×25×13 cm) containing water. 10 days later, the seedlings were transferred into the solution containing 15% PEG for one day, and then 25% PEG for two days. The phenotypes of transgenic lines and WT were investigated and photographed. Three replicates (*n* = 3; 5 plants/genotype/replicate) were used in this experiment. (b) Representative pictures showing WT, OX1 and OX9 under drought stress. Water was withheld from 25-day-old WT, OX1 and OX9 plants grown in the same pot (height×top outer diameter = 14.5×19 cm) for 18 days. (c) 23 days after water withholding, the plants suffering from drought were re-watered, and the survived plants were counted after 5 days of re-watering. Data shown are means and standard deviations of three experiments (*n* = 3; 18 plants/genotype/experiment), with asterisks showing statistically significant differences over the WT (***P* < 0.01; Student’s *t*-test).


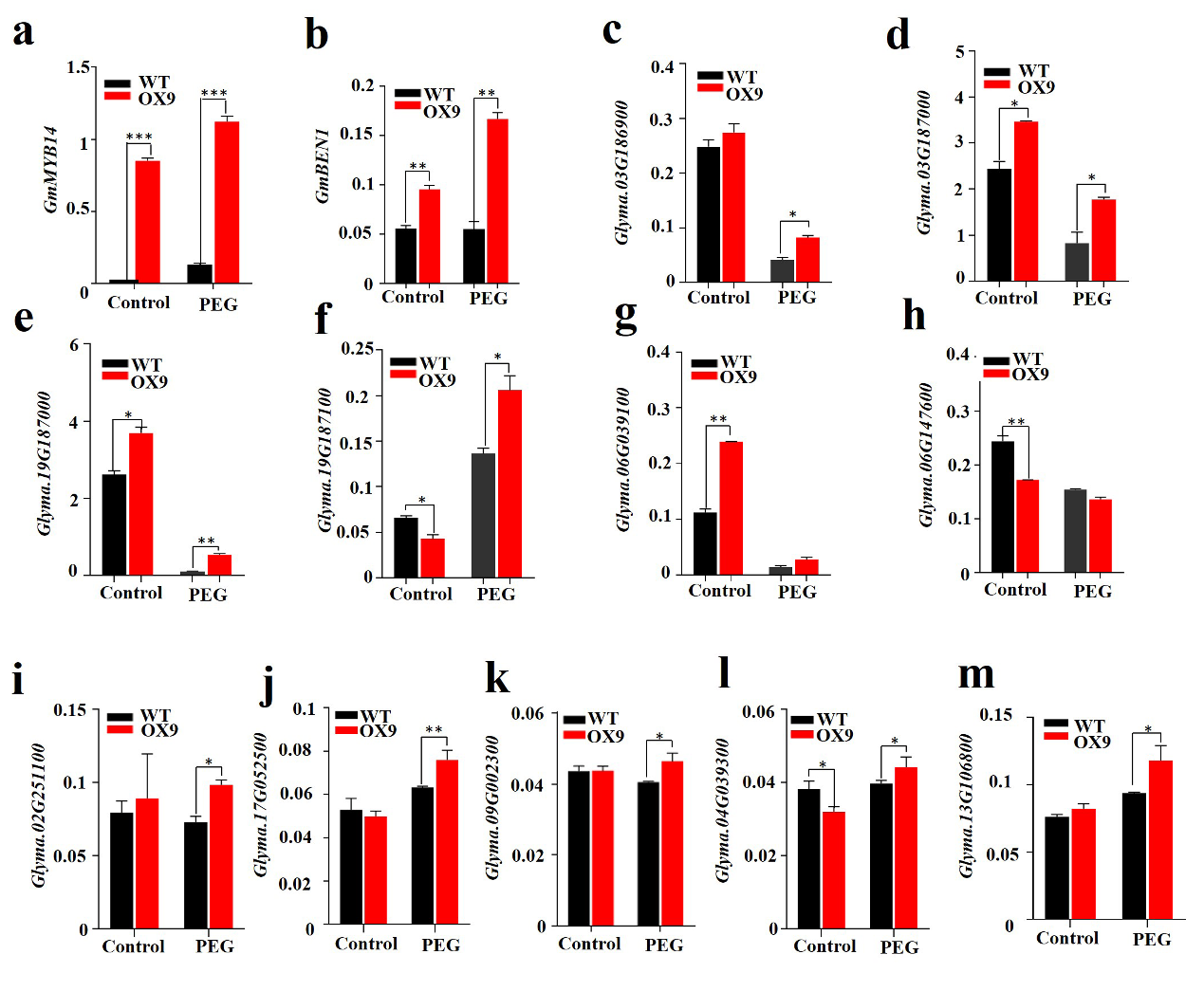


**Figure S5. Expression of** **brassinosteroid (BR)-related genes and drought-related marker genes in wild-type (WT) and OX9 plants exposed to polyethylene glycol (PEG) treatment.** Leaves were harvested from the plants after PEG treatment and used for qRT-PCR analysis. (a) Expression levels of *GmMYB14* in WT and OX9 plants under PEG treatment. (b-h) Selected differentially expressed genes are involved in the biosynthetic or signaling pathway of BR (*GmMYB14/Glyma19g164600, GmBEN1*/*Glyma.18G220600, GmBRI1/Glyma.06G147600*, *GmBKI1/Glyma.06G039100, GmUGT73C5/Glyma.19G187100,* *Glyma.19G187000, Glyma.03G186900* and *Glyma.03G187000*). (i-m) Several drought-related marker genes *Glycine max DELTA-1-PYRROLINE-5-CARBOXYLATE SYNTHASE* (*GmP5CS/Glyma.02G251100* homologous with *AtP5CS*), *G. max ABSCISIC ACID-INSENSITIVE 1* (*GmABI1*/*Glyma.13G106800* and *Glyma.17G052500,* homologous to *AtABI1*), *G. max ABSCISIC ACID3* (*GmABA3*/*Glyma.09G002300*, homologous to *AtABA3*), and *G. max ABA-RESPONSIVE ELEMENTS BINDING FACTOR4* (*GmABF4*/*Glyma.04G039300*, homologous to *AtABF4*) are involved in drought stress responses. Data shown are means and standard deviations (*n* = 3 biological replicates). Statistically significant differences between OX9 and WT plants in terms of gene expression are marked with asterisks (**P* < 0.05, ***P* < 0.01; ****P* < 0.001; Student’s *t*-test).

**
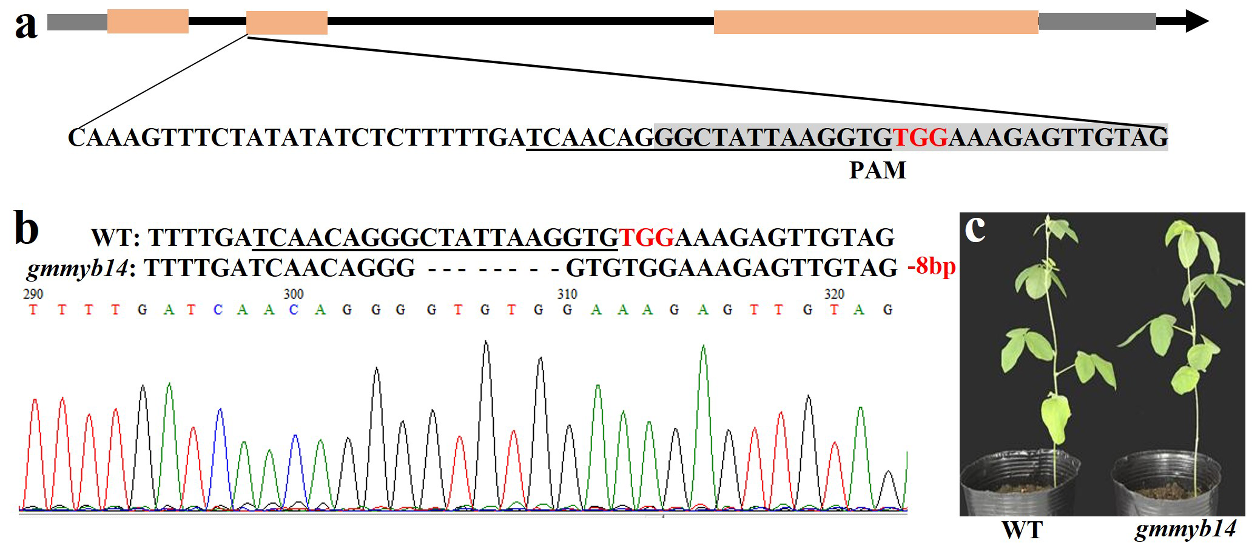
**

**Figure S6.** **CRISPR/Cas9-mediated targeted mutagenesis of *GmMYB14* showed no significant differences in plant architecture of the mutated soybean plants.** (a) Gene structure of *GmMYB14* with one target site. (b) Detailed sequence of the target site of *GmMYB14* in T1 line. (c) Representative picture showing the plant architecture of wild-type (WT) and *gmmyb14* mutant plants grown in an artificial climate chamber (12-h light/12-h dark photoperiod conditions, 28°C) for 28 days.
